# Supplementary material for: Fluorescence molecular optomic signatures improve identification of tumors in head and neck specimens
Source: Front Med Technol. 2023 Feb 15;5:1009638. doi: 10.3389/fmedt.2023.1009638 (PMC9975724; doi:10.3389/fmedt.2023.1009638)
Supplement: Supplementary file 1 [file Datasheet1.docx]

Supplementary Material

# Optomics machine learning scheme details

## Classification pipeline

The supervised machine learning classification pipeline (summarized in the flow chart of **Figure 4B**) was implemented in the Python coding language (v3.8.12) using Scikit-learn (abbreviated “sklearn,” v0.24.2), which is an open-source Python library for machine learning (1). To set up the training-testing paradigm for the whole study, data partitioning (using the “train_test_split” sklearn function) was performed at the beginning of ML pipeline, randomly partitioning images on a slice-level. In the split, 20% of slices were withheld from training. The random seed for selecting the training and testing set indices was user-defined (by setting the “random_state” argument in “train_test_split” sklearn function), such that extreme examples do not appear in the testing set. Given that the dataset contained far more normal than tumor sub-images shown in **Supplementary Table 1**, the normal training sub-images were randomly down-sampled to balance the training set (using the “RandomUnderSampler” function from the imblearn Python package (2), v0.8.0). Optomic features corresponding to training set samples were standardized to z-scores (using the “StandardScaler” sklearn function), and the feature means and standard deviations from the training set standardized the testing set features to minimize data leakage. Optomic features used in model training and testing were selected. The feature selection algorithms identified the subset of *k* features being the highest ranked over all features according to the definition of the feature selection algorithm. All feature selection algorithms involved in this study are from *ITMO_FS* Python library (3), except for minimum redundancy, maximum relevance (MRMR (4)) feature selection algorithm (using the “mrmr” function from the pymrmre Python package (5), v1.0.7). K-nearest neighbors classifier used in this study was set with 50 neighbors to use (n_neighbors=50 for the parameter of “KNeighborsClassifier” sklearn function). AdaBoost classifier in this study was set with the maximum 100 estimators at which boosting is terminated (n_estimators=50 for the parameter of “AdaBoostClassifier” sklearn function). All other classifiers used in this study were with default settings (sklearn function default settings). The choices of ML classifier candidates and feature selection algorithms tested in this study were based on a thorough analysis by Parmar *et al.* (2015) (6). Testing set performance was recorded in the form of classification accuracy.

## Feature extraction using *PyRadiomics*

Optomic feature quantification first requires grey value discretization of the image data. For this study, a fixed bin width of 0.01 was used. Tixier *et al*. found that bin widths that resulted in 30-130 total bins yielded good reproducibility and performance (7). Since the intensity range of all fluorescence images after pre-processing was from 0 to 1, a fixed bin width of 0.01 would produce a hundred total discretized bins for later feature extraction.

Extracted features included a range of first-, second-, and higher order pixel statistics: 18 first-order histogram statistics, 74 second-order features including 23 gray level co-occurrence matrix (GLCM) features, 16 gray level run length matrix (GLRLM) features, 16 gray level size zone matrix (GLSZM) features, 14 gray level dependence matrix (GLDM) features, and five neighboring gray tone difference matrix (NGTDM) features. Furthermore, 1,380 filter-based high-order features (15 filters x 92 features/filter) were included in this study. The fifteen filters were: five Laplacian of Gaussian filters with different image smoothing settings, four wavelet filters by decomposing the image into four frequency sub-band, one square filter, one square root filter, one logarithm filter, one exponential filter, one gradient filter, and one two-dimensional local binary pattern filter.

Extracted features are listed in the appendix at the end of this file, categorized by feature class with feature abbreviations.

# Supplementary Tables and Figures

## Supplementary Tables

Table 1. Tumor and normal sub-image totals in the training set by sub-image size.

| **Tissue type** | **1.81 mm** $\boldsymbol{\times}$ **1.81 mm** | **1.39 mm** $\boldsymbol{\times}$ **1.39 mm** | **0.88 mm** $\boldsymbol{\times}$ **0.88 mm** |
| --- | --- | --- | --- |
| **Tumor** | 2,735 | 4,676 | 11,645 |
| **Normal** | 11,928 | 20,254 | 50,015 |
| **Totals** | 14,663 | 24,930 | 61,660 |

## Supplementary Figures


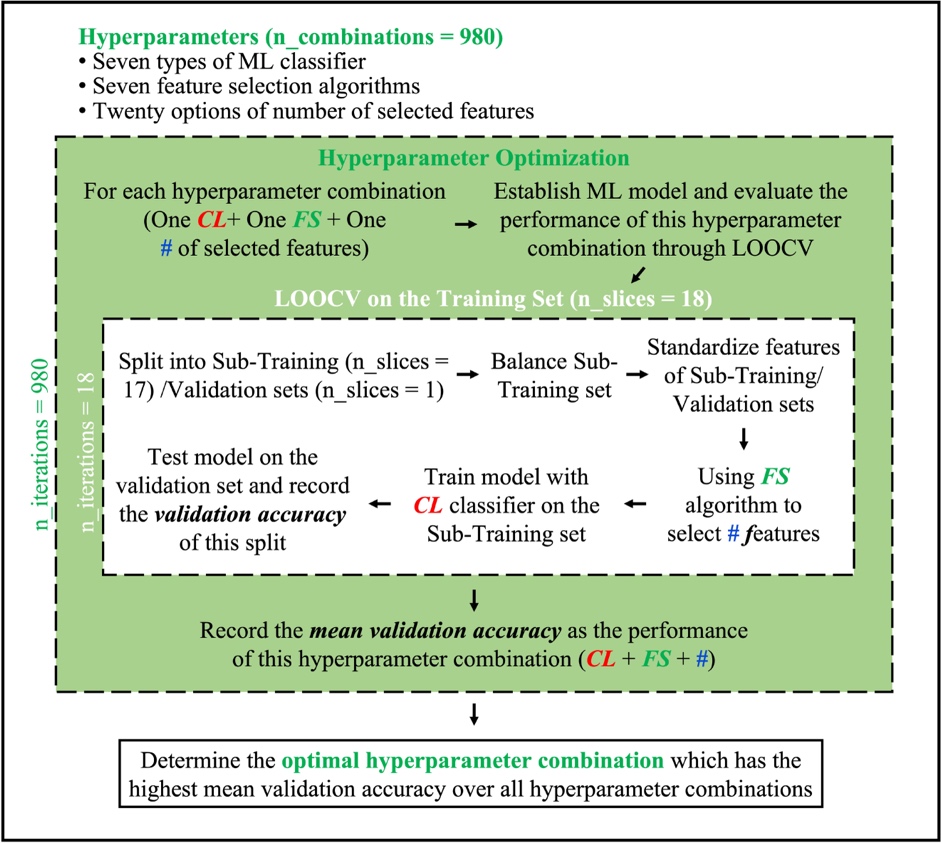


# Supplementary Figure 1. Hyperparameter optimization scheme through Leave-One-Out Cross-Validation (LOOCV). All steps within the dotted box are in iteration. CL = ML classifier, FS = feature selection algorithm, # = number of selected features


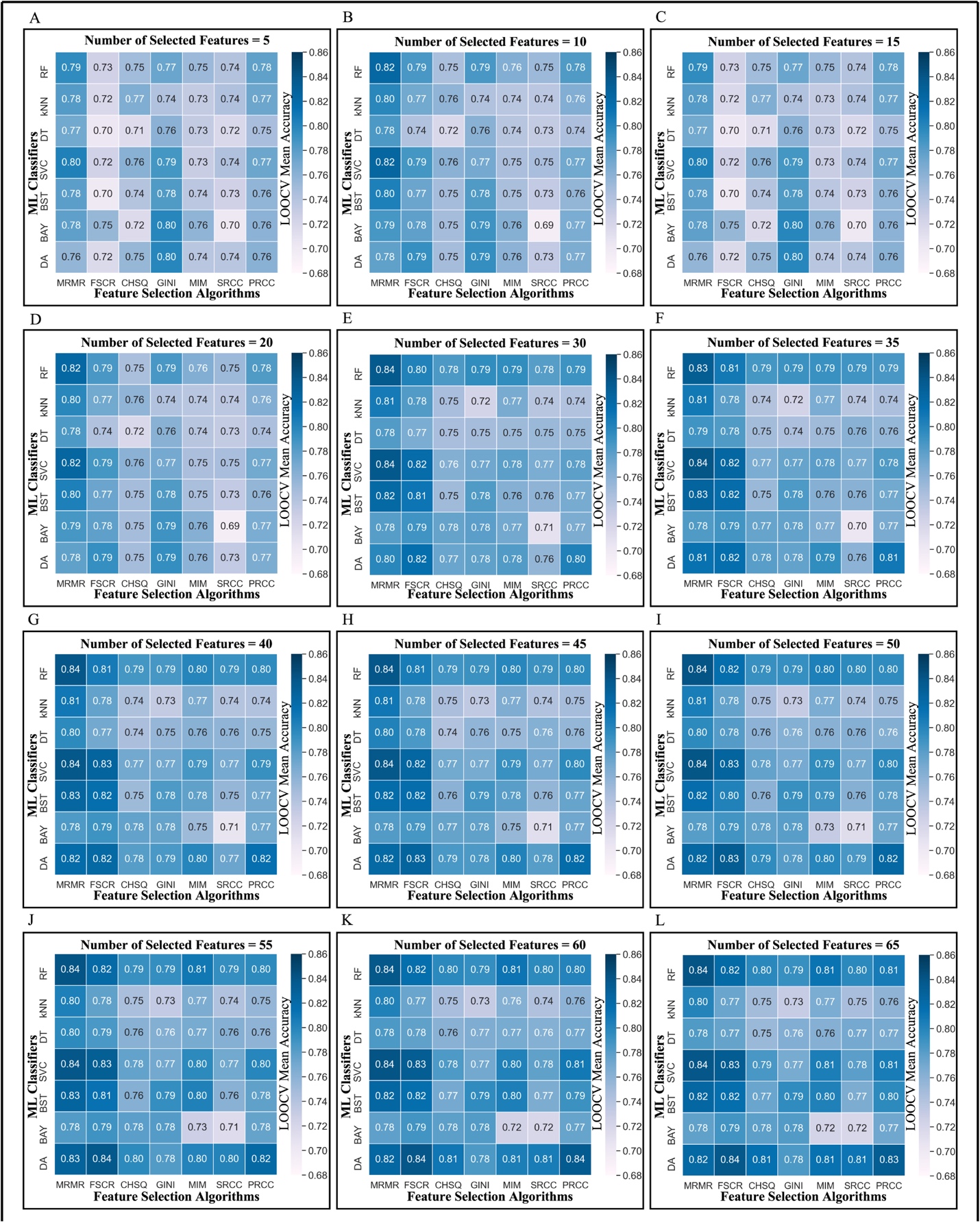


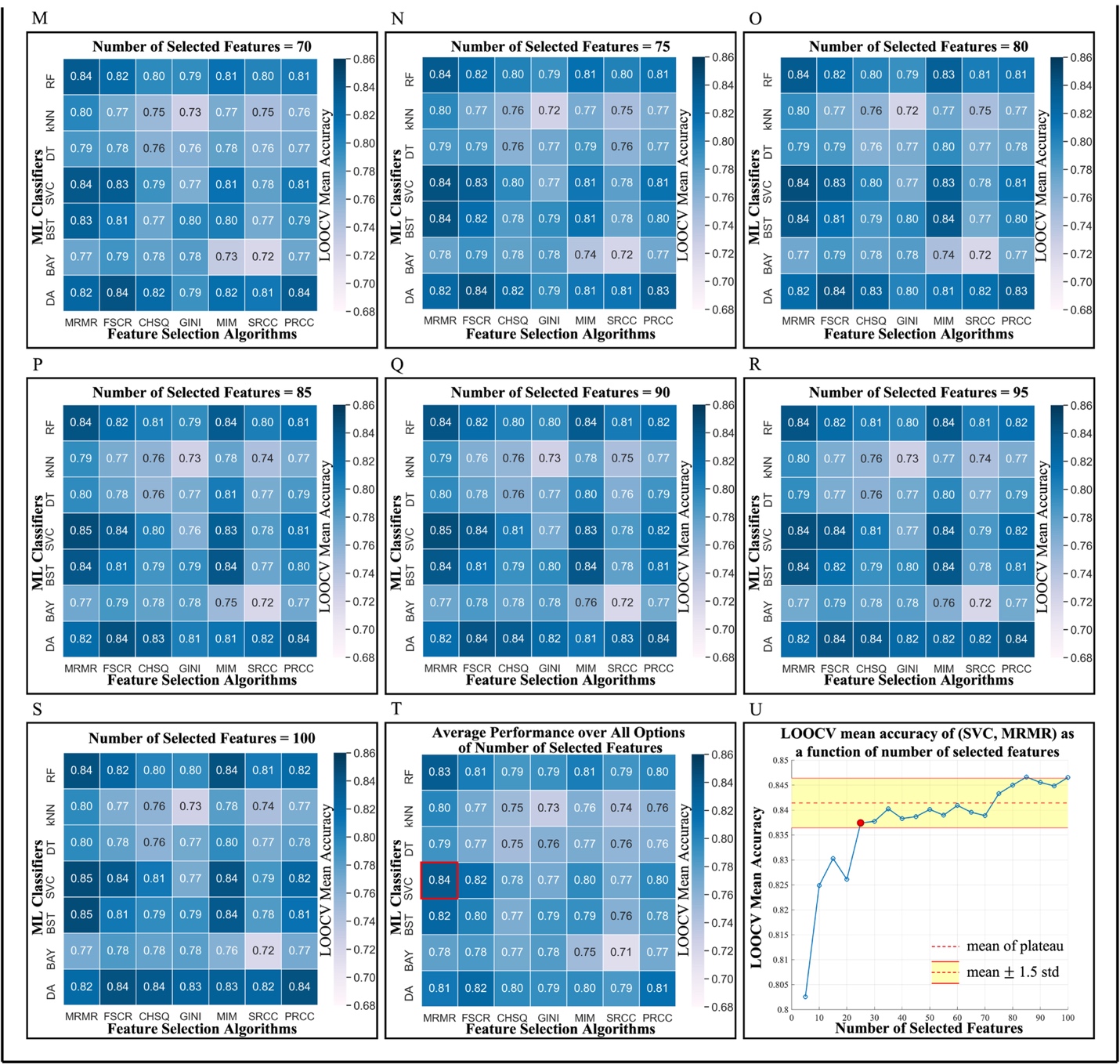


**Supplementary Figure 2.** Results of LOOCV mean accuracy for hyperparameter optimization corresponding to varying numbers of selected features. (A)-(S) Heatmaps of the combination of ML classifiers and feature selection algorithms at different numbers of selected features from 5 to 100 with increment of 5, except for the 25. The heatmap with 25 selected features is shown in Figure 5A in the main body of manuscript. (T) Heatmap that depicts the average LOOCV mean accuracies of the combination of ML classifiers and feature selection algorithms over all options of number of selected features. The combination of SVC ML classifier and MRMR feature selection algorithm labeled in red shows the highest LOOCV mean accuracy in overall, so they were determined as the optimal hyperparameters for ML classifier and feature selection algorithm. (U) LOOCV mean accuracy of the combination of SVC ML classifier and MRMR feature selection algorithm as a function of number of selected features. The feature number at the data point labeled in red was determined as the optimal hyperparameter for number of selected features since it was with the fewest selected features but still in the accuracy tolerance region displayed in yellow.


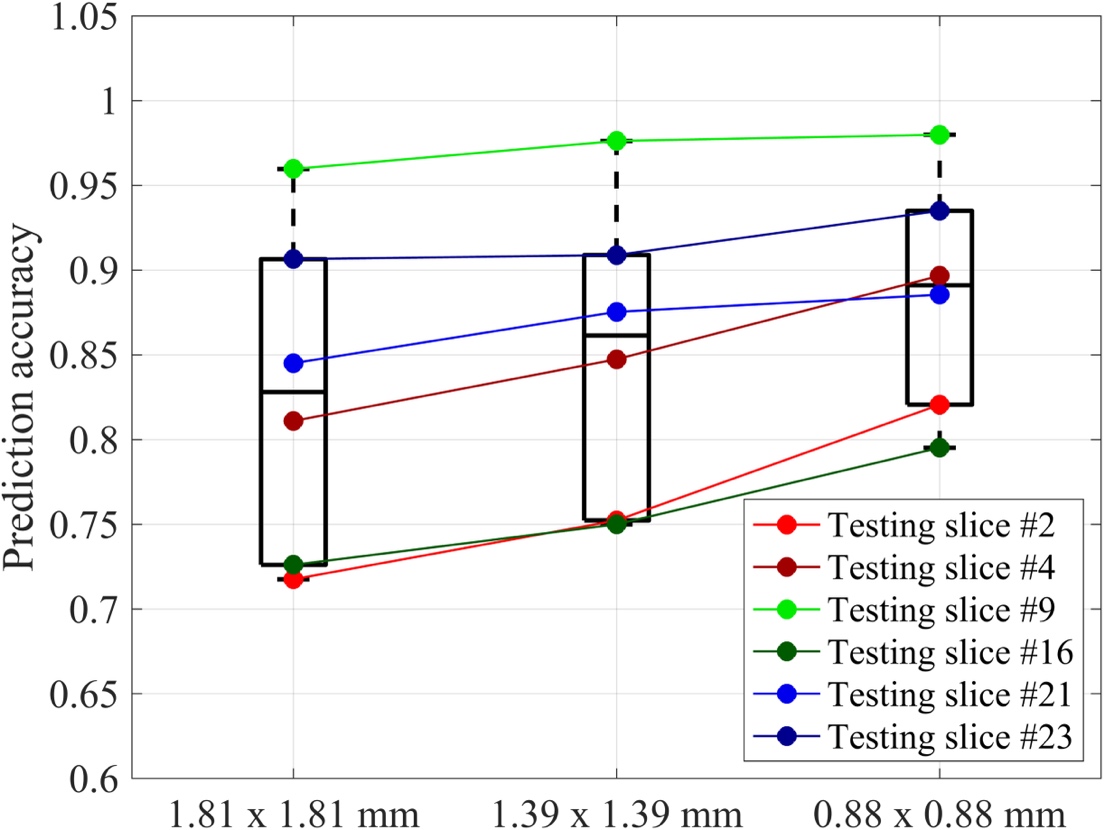


**Supplementary Figure 3.** Prediction performance comparison among the models established based on three sub-image sizes. 1.81 x 1.81 mm acquires the highest prediction accuracy.


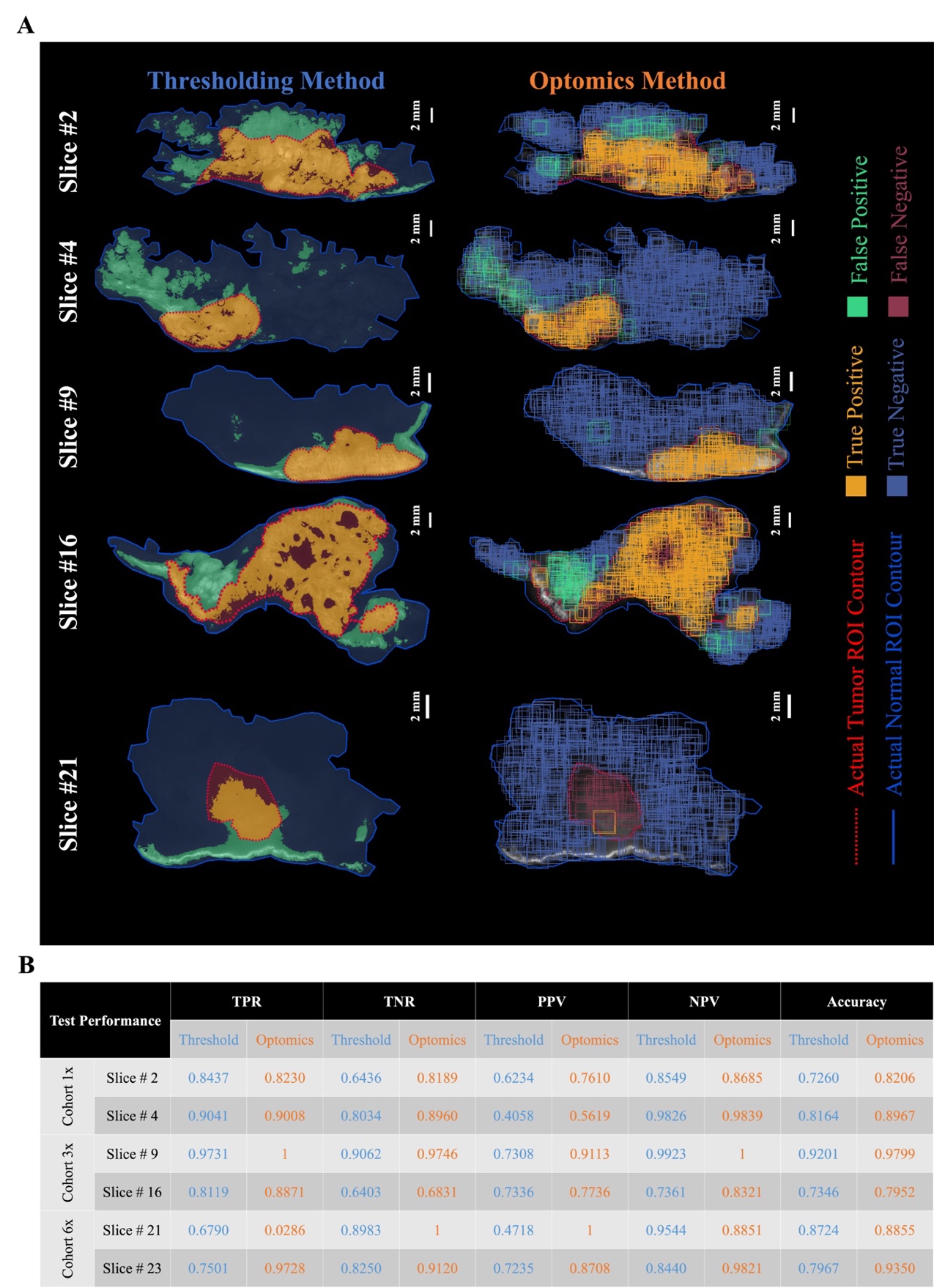


**Supplementary Figure 4.** Comparison of prediction performance of thresholding and optomics `on the other testing slices. (A) Visualization of prediction by two methods on five testing slices respectively. (B) Summary of all prediction performance metrics of two methods.


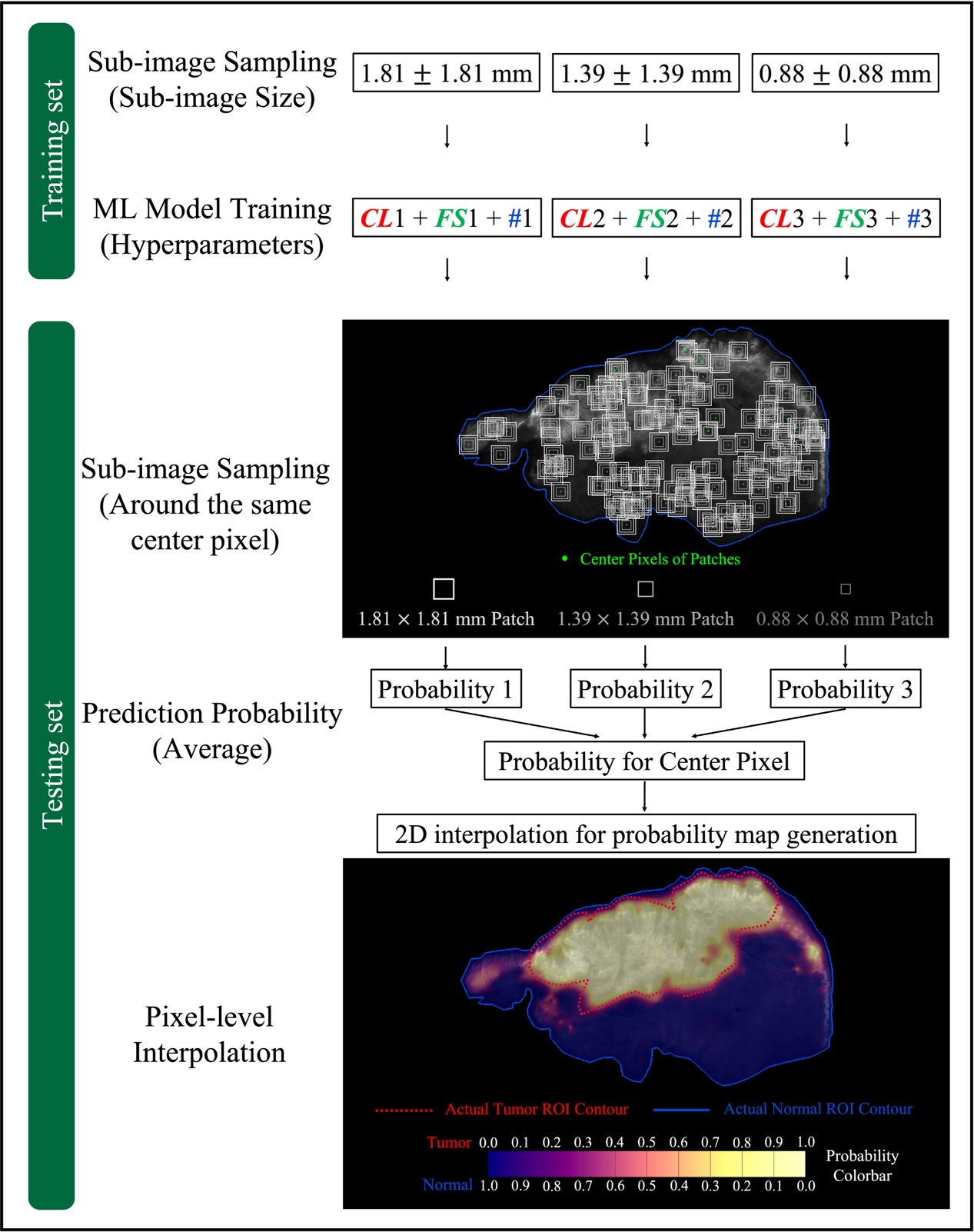


**Supplementary Figure 5.** Workflow of combining three sub-image sizes models to generate probability map.


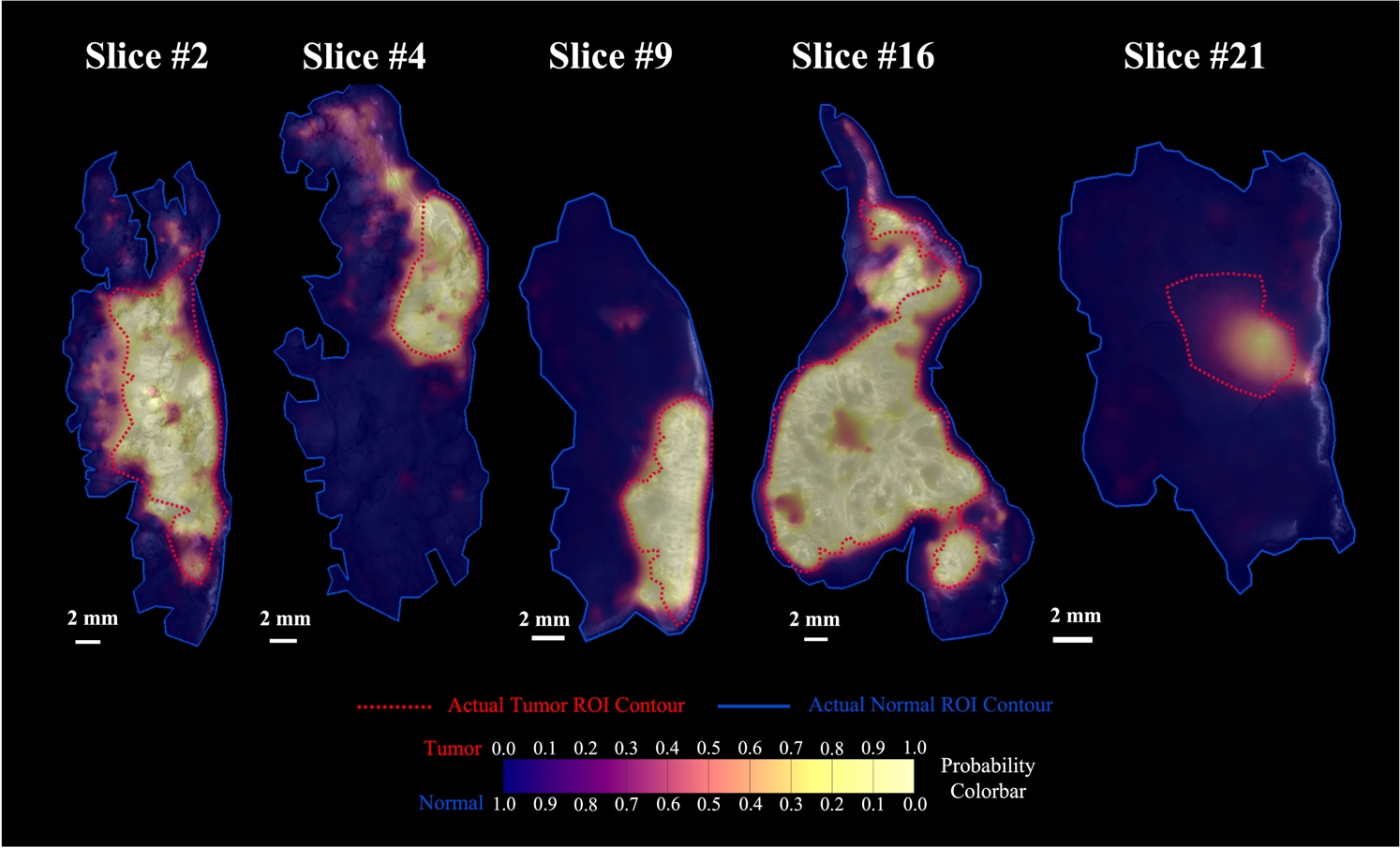


**Supplementary Figure 6.** Optomics method prediction probability maps of the rest five testing slices.


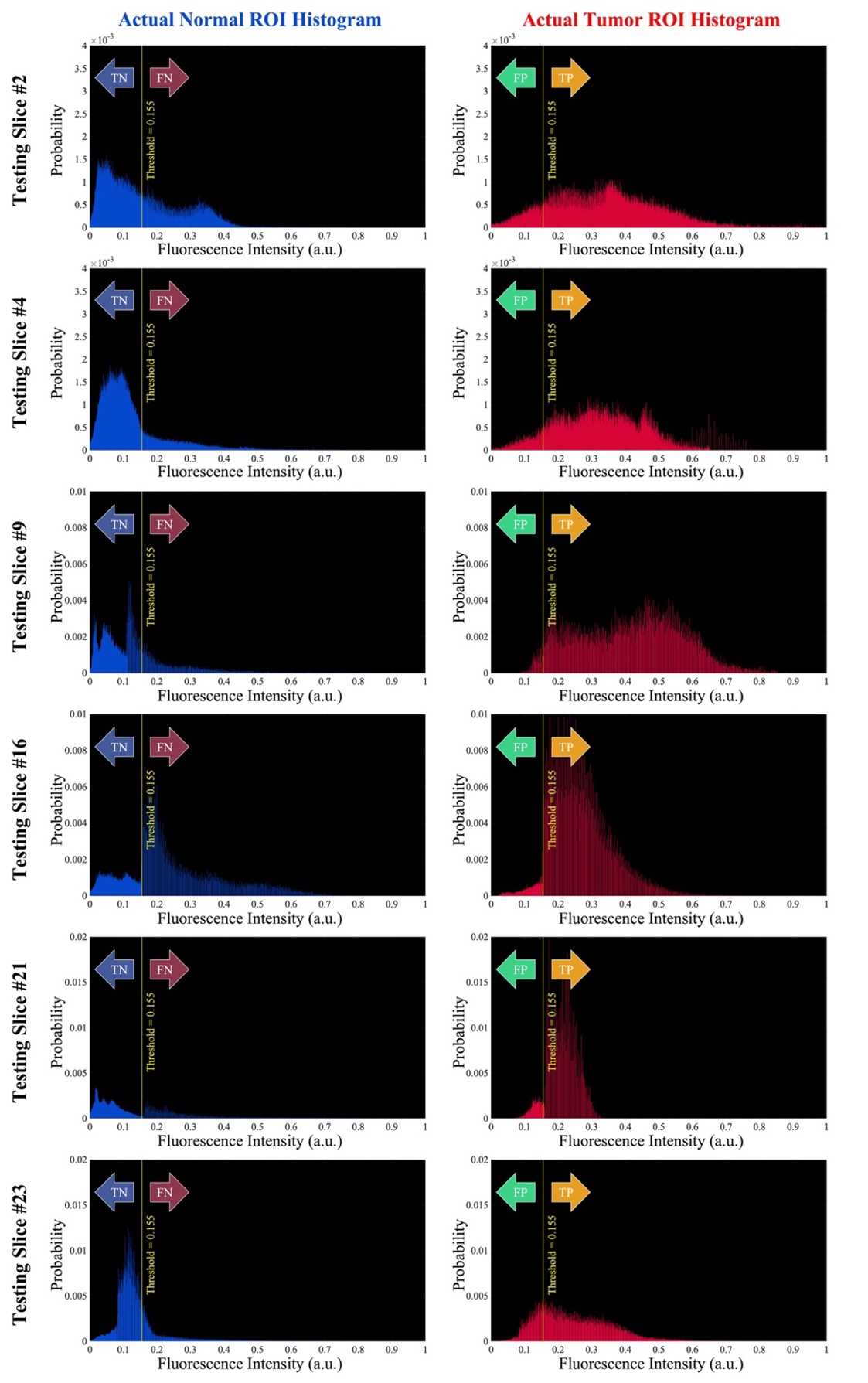


**Supplementary Figure 7.** Illustration of the thresholding method of prediction using pathologist-determined tumor and normal ROI histograms of the testing slices, and corresponding thresholding method prediction correctness illustration.

**Appendix: Optomic Features List**

**First-order histogram statistics:**

1. original first-order 10Percentile
2. original first-order 90Percentile
3. original first-order Energy
4. original first-order Entropy
5. original first-order Interquartile Range
6. original first-order Kurtosis
7. original first-order Maximum
8. original first-order Mean Absolute Deviation
9. original first-order Mean
10. original first-order Median
11. original first-order Minimum
12. original first-order Range
13. original first-order Robust Mean Absolute Deviation
14. original first-order Root Mean Squared
15. original first-order Skewness
16. original first-order Total Energy
17. original first-order Uniformity
18. original first-order Variance

**Second-order features:**

**Gray level co-occurrence matrix (GLCM) features:**

1. original GLCM Autocorrelation
2. original GLCM Cluster Prominence
3. original GLCM Cluster Shade
4. original GLCM Cluster Tendency
5. original GLCM Contrast
6. original GLCM Correlation
7. original GLCM Difference Average
8. original GLCM Difference Entropy
9. original GLCM Difference Variance
10. original GLCM Inverse Difference
11. original GLCM Inverse Difference Moment
12. original GLCM Inverse Difference Moment Normalized
13. original GLCM Inverse Difference Normalized
14. original GLCM Informational Measure of Correlation 1
15. original GLCM Informational Measure of Correlation 2
16. original GLCM Inverse Variance
17. original GLCM Joint Average
18. original GLCM Joint Energy
19. original GLCM Joint Entropy
20. original GLCM MCC
21. original GLCM Maximum Probability
22. original GLCM Sum Entropy
23. original GLCM Sum Squares

**Gray level run length matrix (GLRLM) features:**

1. original GLRLM Gray Level Non-Uniformity
2. original GLRLM Gray Level Non-Uniformity Normalized
3. original GLRLM Gray Level Variance
4. original GLRLM High Gray Level Run Emphasis
5. original GLRLM Long Run Emphasis
6. original GLRLM Long Run High Gray Level Emphasis
7. original GLRLM Long Run Low Gray Level Emphasis
8. original GLRLM Low Gray Level Run Emphasis
9. original GLRLM Run Entropy
10. original GLRLM Run Length Non-Uniformity
11. original GLRLM Run Length Non-Uniformity Normalized
12. original GLRLM Run Percentage
13. original GLRLM Run Variance
14. original GLRLM Short Run Emphasis
15. original GLRLM Short Run High Gray Level Emphasis
16. original GLRLM Short Run Low Gray Level Emphasis

**Gray level size zone matrix (GLSZM) features:**

1. original GLSZM Gray Level Non-Uniformity
2. original GLSZM Gray Level Non-Uniformity Normalized
3. original GLSZM Gray Level Variance
4. original GLSZM High Gray Level Zone Emphasis
5. original GLSZM Large Area Emphasis
6. original GLSZM Large Area High Gray Level Emphasis
7. original GLSZM Large Area Low Gray Level Emphasis
8. original GLSZM Low Gray Level Zone Emphasis
9. original GLSZM Size Zone Non-Uniformity
10. original GLSZM Size Zone Non-Uniformity Normalized
11. original GLSZM Small Area Emphasis
12. original GLSZM Small Area High Gray Level Emphasis
13. original GLSZM Small Area Low Gray Level Emphasis
14. original GLSZM Zone Entropy
15. original GLSZM Zone Percentage
16. original GLSZM Zone Variance

**Gray level dependence matrix (GLDM) features:**

1. original GLDM Dependence Entropy
2. original GLDM Dependence Non-Uniformity
3. original GLDM Dependence Non-Uniformity Normalized
4. original GLDM Dependence Variance
5. original GLDM Gray Level Non-Uniformity
6. original GLDM Gray Level Variance
7. original GLDM High Gray Level Emphasis
8. original GLDM Large Dependence Emphasis
9. original GLDM Large Dependence High Gray Level Emphasis
10. original GLDM Large Dependence Low Gray Level Emphasis
11. original GLDM Low Gray Level Emphasis
12. original GLDM Small Dependence Emphasis
13. original GLDM Small Dependence High Gray Level Emphasis
14. original GLDM Small Dependence Low Gray Level Emphasis

**Neighboring gray tone difference matrix (NGTDM) features:**

1. original NGTDM Busyness
2. original NGTDM Coarseness
3. original NGTDM Complexity
4. original NGTDM Contrast
5. original NGTDM Strength

**Higher-order features (with imposed filters):**

1. log-sigma-0-5-mm-3D first-order 10Percentile
2. log-sigma-0-5-mm-3D first-order 90Percentile
3. log-sigma-0-5-mm-3D first-order Energy
4. log-sigma-0-5-mm-3D first-order Entropy
5. log-sigma-0-5-mm-3D first-order Interquartile Range
6. log-sigma-0-5-mm-3D first-order Kurtosis
7. log-sigma-0-5-mm-3D first-order Maximum
8. log-sigma-0-5-mm-3D first-order Mean Absolute Deviation
9. log-sigma-0-5-mm-3D first-order Mean
10. log-sigma-0-5-mm-3D first-order Median
11. log-sigma-0-5-mm-3D first-order Minimum
12. log-sigma-0-5-mm-3D first-order Range
13. log-sigma-0-5-mm-3D first-order Robust Mean Absolute Deviation
14. log-sigma-0-5-mm-3D first-order Root Mean Squared
15. log-sigma-0-5-mm-3D first-order Skewness
16. log-sigma-0-5-mm-3D first-order Total Energy
17. log-sigma-0-5-mm-3D first-order Uniformity
18. log-sigma-0-5-mm-3D first-order Variance
19. log-sigma-0-5-mm-3D GLCM Autocorrelation
20. log-sigma-0-5-mm-3D GLCM Cluster Prominence
21. log-sigma-0-5-mm-3D GLCM Cluster Shade
22. log-sigma-0-5-mm-3D GLCM Cluster Tendency
23. log-sigma-0-5-mm-3D GLCM Contrast
24. log-sigma-0-5-mm-3D GLCM Correlation
25. log-sigma-0-5-mm-3D GLCM Difference Average
26. log-sigma-0-5-mm-3D GLCM Difference Entropy
27. log-sigma-0-5-mm-3D GLCM Difference Variance
28. log-sigma-0-5-mm-3D GLCM Inverse Difference
29. log-sigma-0-5-mm-3D GLCM Inverse Difference Moment
30. log-sigma-0-5-mm-3D GLCM Inverse Difference Moment Normalized
31. log-sigma-0-5-mm-3D GLCM Inverse Difference Normalized
32. log-sigma-0-5-mm-3D GLCM Informational Measure of Correlation 1
33. log-sigma-0-5-mm-3D GLCM Informational Measure of Correlation 2
34. log-sigma-0-5-mm-3D GLCM Inverse Variance
35. log-sigma-0-5-mm-3D GLCM Joint Average
36. log-sigma-0-5-mm-3D GLCM Joint Energy
37. log-sigma-0-5-mm-3D GLCM Joint Entropy
38. log-sigma-0-5-mm-3D GLCM MCC
39. log-sigma-0-5-mm-3D GLCM Maximum Probability
40. log-sigma-0-5-mm-3D GLCM Sum Entropy
41. log-sigma-0-5-mm-3D GLCM Sum Squares
42. log-sigma-0-5-mm-3D GLRLM Gray Level Non-Uniformity
43. log-sigma-0-5-mm-3D GLRLM Gray Level Non-Uniformity Normalized
44. log-sigma-0-5-mm-3D GLRLM Gray Level Variance
45. log-sigma-0-5-mm-3D GLRLM High Gray Level Run Emphasis
46. log-sigma-0-5-mm-3D GLRLM Long Run Emphasis
47. log-sigma-0-5-mm-3D GLRLM Long Run High Gray Level Emphasis
48. log-sigma-0-5-mm-3D GLRLM Long Run Low Gray Level Emphasis
49. log-sigma-0-5-mm-3D GLRLM Low Gray Level Run Emphasis
50. log-sigma-0-5-mm-3D GLRLM Run Entropy
51. log-sigma-0-5-mm-3D GLRLM Run Length Non-Uniformity
52. log-sigma-0-5-mm-3D GLRLM Run Length Non-Uniformity Normalized
53. log-sigma-0-5-mm-3D GLRLM Run Percentage
54. log-sigma-0-5-mm-3D GLRLM Run Variance
55. log-sigma-0-5-mm-3D GLRLM Short Run Emphasis
56. log-sigma-0-5-mm-3D GLRLM Short Run High Gray Level Emphasis
57. log-sigma-0-5-mm-3D GLRLM Short Run Low Gray Level Emphasis
58. log-sigma-0-5-mm-3D GLSZM Gray Level Non-Uniformity
59. log-sigma-0-5-mm-3D GLSZM Gray Level Non-Uniformity Normalized
60. log-sigma-0-5-mm-3D GLSZM Gray Level Variance
61. log-sigma-0-5-mm-3D GLSZM High Gray Level Zone Emphasis
62. log-sigma-0-5-mm-3D GLSZM Large Area Emphasis
63. log-sigma-0-5-mm-3D GLSZM Large Area High Gray Level Emphasis
64. log-sigma-0-5-mm-3D GLSZM Large Area Low Gray Level Emphasis
65. log-sigma-0-5-mm-3D GLSZM Low Gray Level Zone Emphasis
66. log-sigma-0-5-mm-3D GLSZM Size Zone Non-Uniformity
67. log-sigma-0-5-mm-3D GLSZM Size Zone Non-Uniformity Normalized
68. log-sigma-0-5-mm-3D GLSZM Small Area Emphasis
69. log-sigma-0-5-mm-3D GLSZM Small Area High Gray Level Emphasis
70. log-sigma-0-5-mm-3D GLSZM Small Area Low Gray Level Emphasis
71. log-sigma-0-5-mm-3D GLSZM Zone Entropy
72. log-sigma-0-5-mm-3D GLSZM Zone Percentage
73. log-sigma-0-5-mm-3D GLSZM Zone Variance
74. log-sigma-0-5-mm-3D GLDM Dependence Entropy
75. log-sigma-0-5-mm-3D GLDM Dependence Non-Uniformity
76. log-sigma-0-5-mm-3D GLDM Dependence Non-Uniformity Normalized
77. log-sigma-0-5-mm-3D GLDM Dependence Variance
78. log-sigma-0-5-mm-3D GLDM Gray Level Non-Uniformity
79. log-sigma-0-5-mm-3D GLDM Gray Level Variance
80. log-sigma-0-5-mm-3D GLDM High Gray Level Emphasis
81. log-sigma-0-5-mm-3D GLDM Large Dependence Emphasis
82. log-sigma-0-5-mm-3D GLDM Large Dependence High Gray Level Emphasis
83. log-sigma-0-5-mm-3D GLDM Large Dependence Low Gray Level Emphasis
84. log-sigma-0-5-mm-3D GLDM Low Gray Level Emphasis
85. log-sigma-0-5-mm-3D GLDM Small Dependence Emphasis
86. log-sigma-0-5-mm-3D GLDM Small Dependence High Gray Level Emphasis
87. log-sigma-0-5-mm-3D GLDM Small Dependence Low Gray Level Emphasis
88. log-sigma-0-5-mm-3D NGTDM Busyness
89. log-sigma-0-5-mm-3D NGTDM Coarseness
90. log-sigma-0-5-mm-3D NGTDM Complexity
91. log-sigma-0-5-mm-3D NGTDM Contrast
92. log-sigma-0-5-mm-3D NGTDM Strength
93. log-sigma-1-0-mm-3D first-order 10Percentile
94. log-sigma-1-0-mm-3D first-order 90Percentile
95. log-sigma-1-0-mm-3D first-order Energy
96. log-sigma-1-0-mm-3D first-order Entropy
97. log-sigma-1-0-mm-3D first-order Interquartile Range
98. log-sigma-1-0-mm-3D first-order Kurtosis
99. log-sigma-1-0-mm-3D first-order Maximum
100. log-sigma-1-0-mm-3D first-order Mean Absolute Deviation
101. log-sigma-1-0-mm-3D first-order Mean
102. log-sigma-1-0-mm-3D first-order Median
103. log-sigma-1-0-mm-3D first-order Minimum
104. log-sigma-1-0-mm-3D first-order Range
105. log-sigma-1-0-mm-3D first-order Robust Mean Absolute Deviation
106. log-sigma-1-0-mm-3D first-order Root Mean Squared
107. log-sigma-1-0-mm-3D first-order Skewness
108. log-sigma-1-0-mm-3D first-order Total Energy
109. log-sigma-1-0-mm-3D first-order Uniformity
110. log-sigma-1-0-mm-3D first-order Variance
111. log-sigma-1-0-mm-3D GLCM Autocorrelation
112. log-sigma-1-0-mm-3D GLCM Cluster Prominence
113. log-sigma-1-0-mm-3D GLCM Cluster Shade
114. log-sigma-1-0-mm-3D GLCM Cluster Tendency
115. log-sigma-1-0-mm-3D GLCM Contrast
116. log-sigma-1-0-mm-3D GLCM Correlation
117. log-sigma-1-0-mm-3D GLCM Difference Average
118. log-sigma-1-0-mm-3D GLCM Difference Entropy
119. log-sigma-1-0-mm-3D GLCM Difference Variance
120. log-sigma-1-0-mm-3D GLCM Inverse Difference
121. log-sigma-1-0-mm-3D GLCM Inverse Difference Moment
122. log-sigma-1-0-mm-3D GLCM Inverse Difference Moment Normalized
123. log-sigma-1-0-mm-3D GLCM Inverse Difference Normalized
124. log-sigma-1-0-mm-3D GLCM Informational Measure of Correlation 1
125. log-sigma-1-0-mm-3D GLCM Informational Measure of Correlation 2
126. log-sigma-1-0-mm-3D GLCM Inverse Variance
127. log-sigma-1-0-mm-3D GLCM Joint Average
128. log-sigma-1-0-mm-3D GLCM Joint Energy
129. log-sigma-1-0-mm-3D GLCM Joint Entropy
130. log-sigma-1-0-mm-3D GLCM MCC
131. log-sigma-1-0-mm-3D GLCM Maximum Probability
132. log-sigma-1-0-mm-3D GLCM Sum Entropy
133. log-sigma-1-0-mm-3D GLCM Sum Squares
134. log-sigma-1-0-mm-3D GLRLM Gray Level Non-Uniformity
135. log-sigma-1-0-mm-3D GLRLM Gray Level Non-Uniformity Normalized
136. log-sigma-1-0-mm-3D GLRLM Gray Level Variance
137. log-sigma-1-0-mm-3D GLRLM High Gray Level Run Emphasis
138. log-sigma-1-0-mm-3D GLRLM Long Run Emphasis
139. log-sigma-1-0-mm-3D GLRLM Long Run High Gray Level Emphasis
140. log-sigma-1-0-mm-3D GLRLM Long Run Low Gray Level Emphasis
141. log-sigma-1-0-mm-3D GLRLM Low Gray Level Run Emphasis
142. log-sigma-1-0-mm-3D GLRLM Run Entropy
143. log-sigma-1-0-mm-3D GLRLM Run Length Non-Uniformity
144. log-sigma-1-0-mm-3D GLRLM Run Length Non-Uniformity Normalized
145. log-sigma-1-0-mm-3D GLRLM Run Percentage
146. log-sigma-1-0-mm-3D GLRLM Run Variance
147. log-sigma-1-0-mm-3D GLRLM Short Run Emphasis
148. log-sigma-1-0-mm-3D GLRLM Short Run High Gray Level Emphasis
149. log-sigma-1-0-mm-3D GLRLM Short Run Low Gray Level Emphasis
150. log-sigma-1-0-mm-3D GLSZM Gray Level Non-Uniformity
151. log-sigma-1-0-mm-3D GLSZM Gray Level Non-Uniformity Normalized
152. log-sigma-1-0-mm-3D GLSZM Gray Level Variance
153. log-sigma-1-0-mm-3D GLSZM High Gray Level Zone Emphasis
154. log-sigma-1-0-mm-3D GLSZM Large Area Emphasis
155. log-sigma-1-0-mm-3D GLSZM Large Area High Gray Level Emphasis
156. log-sigma-1-0-mm-3D GLSZM Large Area Low Gray Level Emphasis
157. log-sigma-1-0-mm-3D GLSZM Low Gray Level Zone Emphasis
158. log-sigma-1-0-mm-3D GLSZM Size Zone Non-Uniformity
159. log-sigma-1-0-mm-3D GLSZM Size Zone Non-Uniformity Normalized
160. log-sigma-1-0-mm-3D GLSZM Small Area Emphasis
161. log-sigma-1-0-mm-3D GLSZM Small Area High Gray Level Emphasis
162. log-sigma-1-0-mm-3D GLSZM Small Area Low Gray Level Emphasis
163. log-sigma-1-0-mm-3D GLSZM Zone Entropy
164. log-sigma-1-0-mm-3D GLSZM Zone Percentage
165. log-sigma-1-0-mm-3D GLSZM Zone Variance
166. log-sigma-1-0-mm-3D GLDM Dependence Entropy
167. log-sigma-1-0-mm-3D GLDM Dependence Non-Uniformity
168. log-sigma-1-0-mm-3D GLDM Dependence Non-Uniformity Normalized
169. log-sigma-1-0-mm-3D GLDM Dependence Variance
170. log-sigma-1-0-mm-3D GLDM Gray Level Non-Uniformity
171. log-sigma-1-0-mm-3D GLDM Gray Level Variance
172. log-sigma-1-0-mm-3D GLDM High Gray Level Emphasis
173. log-sigma-1-0-mm-3D GLDM Large Dependence Emphasis
174. log-sigma-1-0-mm-3D GLDM Large Dependence High Gray Level Emphasis
175. log-sigma-1-0-mm-3D GLDM Large Dependence Low Gray Level Emphasis
176. log-sigma-1-0-mm-3D GLDM Low Gray Level Emphasis
177. log-sigma-1-0-mm-3D GLDM Small Dependence Emphasis
178. log-sigma-1-0-mm-3D GLDM Small Dependence High Gray Level Emphasis
179. log-sigma-1-0-mm-3D GLDM Small Dependence Low Gray Level Emphasis
180. log-sigma-1-0-mm-3D NGTDM Busyness
181. log-sigma-1-0-mm-3D NGTDM Coarseness
182. log-sigma-1-0-mm-3D NGTDM Complexity
183. log-sigma-1-0-mm-3D NGTDM Contrast
184. log-sigma-1-0-mm-3D NGTDM Strength
185. log-sigma-1-5-mm-3D first-order 10Percentile
186. log-sigma-1-5-mm-3D first-order 90Percentile
187. log-sigma-1-5-mm-3D first-order Energy
188. log-sigma-1-5-mm-3D first-order Entropy
189. log-sigma-1-5-mm-3D first-order Interquartile Range
190. log-sigma-1-5-mm-3D first-order Kurtosis
191. log-sigma-1-5-mm-3D first-order Maximum
192. log-sigma-1-5-mm-3D first-order Mean Absolute Deviation
193. log-sigma-1-5-mm-3D first-order Mean
194. log-sigma-1-5-mm-3D first-order Median
195. log-sigma-1-5-mm-3D first-order Minimum
196. log-sigma-1-5-mm-3D first-order Range
197. log-sigma-1-5-mm-3D first-order Robust Mean Absolute Deviation
198. log-sigma-1-5-mm-3D first-order Root Mean Squared
199. log-sigma-1-5-mm-3D first-order Skewness
200. log-sigma-1-5-mm-3D first-order Total Energy
201. log-sigma-1-5-mm-3D first-order Uniformity
202. log-sigma-1-5-mm-3D first-order Variance
203. log-sigma-1-5-mm-3D GLCM Autocorrelation
204. log-sigma-1-5-mm-3D GLCM Cluster Prominence
205. log-sigma-1-5-mm-3D GLCM Cluster Shade
206. log-sigma-1-5-mm-3D GLCM Cluster Tendency
207. log-sigma-1-5-mm-3D GLCM Contrast
208. log-sigma-1-5-mm-3D GLCM Correlation
209. log-sigma-1-5-mm-3D GLCM Difference Average
210. log-sigma-1-5-mm-3D GLCM Difference Entropy
211. log-sigma-1-5-mm-3D GLCM Difference Variance
212. log-sigma-1-5-mm-3D GLCM Inverse Difference
213. log-sigma-1-5-mm-3D GLCM Inverse Difference Moment
214. log-sigma-1-5-mm-3D GLCM Inverse Difference Moment Normalized
215. log-sigma-1-5-mm-3D GLCM Inverse Difference Normalized
216. log-sigma-1-5-mm-3D GLCM Informational Measure of Correlation 1
217. log-sigma-1-5-mm-3D GLCM Informational Measure of Correlation 2
218. log-sigma-1-5-mm-3D GLCM Inverse Variance
219. log-sigma-1-5-mm-3D GLCM Joint Average
220. log-sigma-1-5-mm-3D GLCM Joint Energy
221. log-sigma-1-5-mm-3D GLCM Joint Entropy
222. log-sigma-1-5-mm-3D GLCM MCC
223. log-sigma-1-5-mm-3D GLCM Maximum Probability
224. log-sigma-1-5-mm-3D GLCM Sum Entropy
225. log-sigma-1-5-mm-3D GLCM Sum Squares
226. log-sigma-1-5-mm-3D GLRLM Gray Level Non-Uniformity
227. log-sigma-1-5-mm-3D GLRLM Gray Level Non-Uniformity Normalized
228. log-sigma-1-5-mm-3D GLRLM Gray Level Variance
229. log-sigma-1-5-mm-3D GLRLM High Gray Level Run Emphasis
230. log-sigma-1-5-mm-3D GLRLM Long Run Emphasis
231. log-sigma-1-5-mm-3D GLRLM Long Run High Gray Level Emphasis
232. log-sigma-1-5-mm-3D GLRLM Long Run Low Gray Level Emphasis
233. log-sigma-1-5-mm-3D GLRLM Low Gray Level Run Emphasis
234. log-sigma-1-5-mm-3D GLRLM Run Entropy
235. log-sigma-1-5-mm-3D GLRLM Run Length Non-Uniformity
236. log-sigma-1-5-mm-3D GLRLM Run Length Non-Uniformity Normalized
237. log-sigma-1-5-mm-3D GLRLM Run Percentage
238. log-sigma-1-5-mm-3D GLRLM Run Variance
239. log-sigma-1-5-mm-3D GLRLM Short Run Emphasis
240. log-sigma-1-5-mm-3D GLRLM Short Run High Gray Level Emphasis
241. log-sigma-1-5-mm-3D GLRLM Short Run Low Gray Level Emphasis
242. log-sigma-1-5-mm-3D GLSZM Gray Level Non-Uniformity
243. log-sigma-1-5-mm-3D GLSZM Gray Level Non-Uniformity Normalized
244. log-sigma-1-5-mm-3D GLSZM Gray Level Variance
245. log-sigma-1-5-mm-3D GLSZM High Gray Level Zone Emphasis
246. log-sigma-1-5-mm-3D GLSZM Large Area Emphasis
247. log-sigma-1-5-mm-3D GLSZM Large Area High Gray Level Emphasis
248. log-sigma-1-5-mm-3D GLSZM Large Area Low Gray Level Emphasis
249. log-sigma-1-5-mm-3D GLSZM Low Gray Level Zone Emphasis
250. log-sigma-1-5-mm-3D GLSZM Size Zone Non-Uniformity
251. log-sigma-1-5-mm-3D GLSZM Size Zone Non-Uniformity Normalized
252. log-sigma-1-5-mm-3D GLSZM Small Area Emphasis
253. log-sigma-1-5-mm-3D GLSZM Small Area High Gray Level Emphasis
254. log-sigma-1-5-mm-3D GLSZM Small Area Low Gray Level Emphasis
255. log-sigma-1-5-mm-3D GLSZM Zone Entropy
256. log-sigma-1-5-mm-3D GLSZM Zone Percentage
257. log-sigma-1-5-mm-3D GLSZM Zone Variance
258. log-sigma-1-5-mm-3D GLDM Dependence Entropy
259. log-sigma-1-5-mm-3D GLDM Dependence Non-Uniformity
260. log-sigma-1-5-mm-3D GLDM Dependence Non-Uniformity Normalized
261. log-sigma-1-5-mm-3D GLDM Dependence Variance
262. log-sigma-1-5-mm-3D GLDM Gray Level Non-Uniformity
263. log-sigma-1-5-mm-3D GLDM Gray Level Variance
264. log-sigma-1-5-mm-3D GLDM High Gray Level Emphasis
265. log-sigma-1-5-mm-3D GLDM Large Dependence Emphasis
266. log-sigma-1-5-mm-3D GLDM Large Dependence High Gray Level Emphasis
267. log-sigma-1-5-mm-3D GLDM Large Dependence Low Gray Level Emphasis
268. log-sigma-1-5-mm-3D GLDM Low Gray Level Emphasis
269. log-sigma-1-5-mm-3D GLDM Small Dependence Emphasis
270. log-sigma-1-5-mm-3D GLDM Small Dependence High Gray Level Emphasis
271. log-sigma-1-5-mm-3D GLDM Small Dependence Low Gray Level Emphasis
272. log-sigma-1-5-mm-3D NGTDM Busyness
273. log-sigma-1-5-mm-3D NGTDM Coarseness
274. log-sigma-1-5-mm-3D NGTDM Complexity
275. log-sigma-1-5-mm-3D NGTDM Contrast
276. log-sigma-1-5-mm-3D NGTDM Strength
277. log-sigma-2-0-mm-3D first-order 10Percentile
278. log-sigma-2-0-mm-3D first-order 90Percentile
279. log-sigma-2-0-mm-3D first-order Energy
280. log-sigma-2-0-mm-3D first-order Entropy
281. log-sigma-2-0-mm-3D first-order Interquartile Range
282. log-sigma-2-0-mm-3D first-order Kurtosis
283. log-sigma-2-0-mm-3D first-order Maximum
284. log-sigma-2-0-mm-3D first-order Mean Absolute Deviation
285. log-sigma-2-0-mm-3D first-order Mean
286. log-sigma-2-0-mm-3D first-order Median
287. log-sigma-2-0-mm-3D first-order Minimum
288. log-sigma-2-0-mm-3D first-order Range
289. log-sigma-2-0-mm-3D first-order Robust Mean Absolute Deviation
290. log-sigma-2-0-mm-3D first-order Root Mean Squared
291. log-sigma-2-0-mm-3D first-order Skewness
292. log-sigma-2-0-mm-3D first-order Total Energy
293. log-sigma-2-0-mm-3D first-order Uniformity
294. log-sigma-2-0-mm-3D first-order Variance
295. log-sigma-2-0-mm-3D GLCM Autocorrelation
296. log-sigma-2-0-mm-3D GLCM Cluster Prominence
297. log-sigma-2-0-mm-3D GLCM Cluster Shade
298. log-sigma-2-0-mm-3D GLCM Cluster Tendency
299. log-sigma-2-0-mm-3D GLCM Contrast
300. log-sigma-2-0-mm-3D GLCM Correlation
301. log-sigma-2-0-mm-3D GLCM Difference Average
302. log-sigma-2-0-mm-3D GLCM Difference Entropy
303. log-sigma-2-0-mm-3D GLCM Difference Variance
304. log-sigma-2-0-mm-3D GLCM Inverse Difference
305. log-sigma-2-0-mm-3D GLCM Inverse Difference Moment
306. log-sigma-2-0-mm-3D GLCM Inverse Difference Moment Normalized
307. log-sigma-2-0-mm-3D GLCM Inverse Difference Normalized
308. log-sigma-2-0-mm-3D GLCM Informational Measure of Correlation 1
309. log-sigma-2-0-mm-3D GLCM Informational Measure of Correlation 2
310. log-sigma-2-0-mm-3D GLCM Inverse Variance
311. log-sigma-2-0-mm-3D GLCM Joint Average
312. log-sigma-2-0-mm-3D GLCM Joint Energy
313. log-sigma-2-0-mm-3D GLCM Joint Entropy
314. log-sigma-2-0-mm-3D GLCM MCC
315. log-sigma-2-0-mm-3D GLCM Maximum Probability
316. log-sigma-2-0-mm-3D GLCM Sum Entropy
317. log-sigma-2-0-mm-3D GLCM Sum Squares
318. log-sigma-2-0-mm-3D GLRLM Gray Level Non-Uniformity
319. log-sigma-2-0-mm-3D GLRLM Gray Level Non-Uniformity Normalized
320. log-sigma-2-0-mm-3D GLRLM Gray Level Variance
321. log-sigma-2-0-mm-3D GLRLM High Gray Level Run Emphasis
322. log-sigma-2-0-mm-3D GLRLM Long Run Emphasis
323. log-sigma-2-0-mm-3D GLRLM Long Run High Gray Level Emphasis
324. log-sigma-2-0-mm-3D GLRLM Long Run Low Gray Level Emphasis
325. log-sigma-2-0-mm-3D GLRLM Low Gray Level Run Emphasis
326. log-sigma-2-0-mm-3D GLRLM Run Entropy
327. log-sigma-2-0-mm-3D GLRLM Run Length Non-Uniformity
328. log-sigma-2-0-mm-3D GLRLM Run Length Non-Uniformity Normalized
329. log-sigma-2-0-mm-3D GLRLM Run Percentage
330. log-sigma-2-0-mm-3D GLRLM Run Variance
331. log-sigma-2-0-mm-3D GLRLM Short Run Emphasis
332. log-sigma-2-0-mm-3D GLRLM Short Run High Gray Level Emphasis
333. log-sigma-2-0-mm-3D GLRLM Short Run Low Gray Level Emphasis
334. log-sigma-2-0-mm-3D GLSZM Gray Level Non-Uniformity
335. log-sigma-2-0-mm-3D GLSZM Gray Level Non-Uniformity Normalized
336. log-sigma-2-0-mm-3D GLSZM Gray Level Variance
337. log-sigma-2-0-mm-3D GLSZM High Gray Level Zone Emphasis
338. log-sigma-2-0-mm-3D GLSZM Large Area Emphasis
339. log-sigma-2-0-mm-3D GLSZM Large Area High Gray Level Emphasis
340. log-sigma-2-0-mm-3D GLSZM Large Area Low Gray Level Emphasis
341. log-sigma-2-0-mm-3D GLSZM Low Gray Level Zone Emphasis
342. log-sigma-2-0-mm-3D GLSZM Size Zone Non-Uniformity
343. log-sigma-2-0-mm-3D GLSZM Size Zone Non-Uniformity Normalized
344. log-sigma-2-0-mm-3D GLSZM Small Area Emphasis
345. log-sigma-2-0-mm-3D GLSZM Small Area High Gray Level Emphasis
346. log-sigma-2-0-mm-3D GLSZM Small Area Low Gray Level Emphasis
347. log-sigma-2-0-mm-3D GLSZM Zone Entropy
348. log-sigma-2-0-mm-3D GLSZM Zone Percentage
349. log-sigma-2-0-mm-3D GLSZM Zone Variance
350. log-sigma-2-0-mm-3D GLDM Dependence Entropy
351. log-sigma-2-0-mm-3D GLDM Dependence Non-Uniformity
352. log-sigma-2-0-mm-3D GLDM Dependence Non-Uniformity Normalized
353. log-sigma-2-0-mm-3D GLDM Dependence Variance
354. log-sigma-2-0-mm-3D GLDM Gray Level Non-Uniformity
355. log-sigma-2-0-mm-3D GLDM Gray Level Variance
356. log-sigma-2-0-mm-3D GLDM High Gray Level Emphasis
357. log-sigma-2-0-mm-3D GLDM Large Dependence Emphasis
358. log-sigma-2-0-mm-3D GLDM Large Dependence High Gray Level Emphasis
359. log-sigma-2-0-mm-3D GLDM Large Dependence Low Gray Level Emphasis
360. log-sigma-2-0-mm-3D GLDM Low Gray Level Emphasis
361. log-sigma-2-0-mm-3D GLDM Small Dependence Emphasis
362. log-sigma-2-0-mm-3D GLDM Small Dependence High Gray Level Emphasis
363. log-sigma-2-0-mm-3D GLDM Small Dependence Low Gray Level Emphasis
364. log-sigma-2-0-mm-3D NGTDM Busyness
365. log-sigma-2-0-mm-3D NGTDM Coarseness
366. log-sigma-2-0-mm-3D NGTDM Complexity
367. log-sigma-2-0-mm-3D NGTDM Contrast
368. log-sigma-2-0-mm-3D NGTDM Strength
369. log-sigma-2-5-mm-3D first-order 10Percentile
370. log-sigma-2-5-mm-3D first-order 90Percentile
371. log-sigma-2-5-mm-3D first-order Energy
372. log-sigma-2-5-mm-3D first-order Entropy
373. log-sigma-2-5-mm-3D first-order Interquartile Range
374. log-sigma-2-5-mm-3D first-order Kurtosis
375. log-sigma-2-5-mm-3D first-order Maximum
376. log-sigma-2-5-mm-3D first-order Mean Absolute Deviation
377. log-sigma-2-5-mm-3D first-order Mean
378. log-sigma-2-5-mm-3D first-order Median
379. log-sigma-2-5-mm-3D first-order Minimum
380. log-sigma-2-5-mm-3D first-order Range
381. log-sigma-2-5-mm-3D first-order Robust Mean Absolute Deviation
382. log-sigma-2-5-mm-3D first-order Root Mean Squared
383. log-sigma-2-5-mm-3D first-order Skewness
384. log-sigma-2-5-mm-3D first-order Total Energy
385. log-sigma-2-5-mm-3D first-order Uniformity
386. log-sigma-2-5-mm-3D first-order Variance
387. log-sigma-2-5-mm-3D GLCM Autocorrelation
388. log-sigma-2-5-mm-3D GLCM Cluster Prominence
389. log-sigma-2-5-mm-3D GLCM Cluster Shade
390. log-sigma-2-5-mm-3D GLCM Cluster Tendency
391. log-sigma-2-5-mm-3D GLCM Contrast
392. log-sigma-2-5-mm-3D GLCM Correlation
393. log-sigma-2-5-mm-3D GLCM Difference Average
394. log-sigma-2-5-mm-3D GLCM Difference Entropy
395. log-sigma-2-5-mm-3D GLCM Difference Variance
396. log-sigma-2-5-mm-3D GLCM Inverse Difference
397. log-sigma-2-5-mm-3D GLCM Inverse Difference Moment
398. log-sigma-2-5-mm-3D GLCM Inverse Difference Moment Normalized
399. log-sigma-2-5-mm-3D GLCM Inverse Difference Normalized
400. log-sigma-2-5-mm-3D GLCM Informational Measure of Correlation 1
401. log-sigma-2-5-mm-3D GLCM Informational Measure of Correlation 2
402. log-sigma-2-5-mm-3D GLCM Inverse Variance
403. log-sigma-2-5-mm-3D GLCM Joint Average
404. log-sigma-2-5-mm-3D GLCM Joint Energy
405. log-sigma-2-5-mm-3D GLCM Joint Entropy
406. log-sigma-2-5-mm-3D GLCM MCC
407. log-sigma-2-5-mm-3D GLCM Maximum Probability
408. log-sigma-2-5-mm-3D GLCM Sum Entropy
409. log-sigma-2-5-mm-3D GLCM Sum Squares
410. log-sigma-2-5-mm-3D GLRLM Gray Level Non-Uniformity
411. log-sigma-2-5-mm-3D GLRLM Gray Level Non-Uniformity Normalized
412. log-sigma-2-5-mm-3D GLRLM Gray Level Variance
413. log-sigma-2-5-mm-3D GLRLM High Gray Level Run Emphasis
414. log-sigma-2-5-mm-3D GLRLM Long Run Emphasis
415. log-sigma-2-5-mm-3D GLRLM Long Run High Gray Level Emphasis
416. log-sigma-2-5-mm-3D GLRLM Long Run Low Gray Level Emphasis
417. log-sigma-2-5-mm-3D GLRLM Low Gray Level Run Emphasis
418. log-sigma-2-5-mm-3D GLRLM Run Entropy
419. log-sigma-2-5-mm-3D GLRLM Run Length Non-Uniformity
420. log-sigma-2-5-mm-3D GLRLM Run Length Non-Uniformity Normalized
421. log-sigma-2-5-mm-3D GLRLM Run Percentage
422. log-sigma-2-5-mm-3D GLRLM Run Variance
423. log-sigma-2-5-mm-3D GLRLM Short Run Emphasis
424. log-sigma-2-5-mm-3D GLRLM Short Run High Gray Level Emphasis
425. log-sigma-2-5-mm-3D GLRLM Short Run Low Gray Level Emphasis
426. log-sigma-2-5-mm-3D GLSZM Gray Level Non-Uniformity
427. log-sigma-2-5-mm-3D GLSZM Gray Level Non-Uniformity Normalized
428. log-sigma-2-5-mm-3D GLSZM Gray Level Variance
429. log-sigma-2-5-mm-3D GLSZM High Gray Level Zone Emphasis
430. log-sigma-2-5-mm-3D GLSZM Large Area Emphasis
431. log-sigma-2-5-mm-3D GLSZM Large Area High Gray Level Emphasis
432. log-sigma-2-5-mm-3D GLSZM Large Area Low Gray Level Emphasis
433. log-sigma-2-5-mm-3D GLSZM Low Gray Level Zone Emphasis
434. log-sigma-2-5-mm-3D GLSZM Size Zone Non-Uniformity
435. log-sigma-2-5-mm-3D GLSZM Size Zone Non-Uniformity Normalized
436. log-sigma-2-5-mm-3D GLSZM Small Area Emphasis
437. log-sigma-2-5-mm-3D GLSZM Small Area High Gray Level Emphasis
438. log-sigma-2-5-mm-3D GLSZM Small Area Low Gray Level Emphasis
439. log-sigma-2-5-mm-3D GLSZM Zone Entropy
440. log-sigma-2-5-mm-3D GLSZM Zone Percentage
441. log-sigma-2-5-mm-3D GLSZM Zone Variance
442. log-sigma-2-5-mm-3D GLDM Dependence Entropy
443. log-sigma-2-5-mm-3D GLDM Dependence Non-Uniformity
444. log-sigma-2-5-mm-3D GLDM Dependence Non-Uniformity Normalized
445. log-sigma-2-5-mm-3D GLDM Dependence Variance
446. log-sigma-2-5-mm-3D GLDM Gray Level Non-Uniformity
447. log-sigma-2-5-mm-3D GLDM Gray Level Variance
448. log-sigma-2-5-mm-3D GLDM High Gray Level Emphasis
449. log-sigma-2-5-mm-3D GLDM Large Dependence Emphasis
450. log-sigma-2-5-mm-3D GLDM Large Dependence High Gray Level Emphasis
451. log-sigma-2-5-mm-3D GLDM Large Dependence Low Gray Level Emphasis
452. log-sigma-2-5-mm-3D GLDM Low Gray Level Emphasis
453. log-sigma-2-5-mm-3D GLDM Small Dependence Emphasis
454. log-sigma-2-5-mm-3D GLDM Small Dependence High Gray Level Emphasis
455. log-sigma-2-5-mm-3D GLDM Small Dependence Low Gray Level Emphasis
456. log-sigma-2-5-mm-3D NGTDM Busyness
457. log-sigma-2-5-mm-3D NGTDM Coarseness
458. log-sigma-2-5-mm-3D NGTDM Complexity
459. log-sigma-2-5-mm-3D NGTDM Contrast
460. log-sigma-2-5-mm-3D NGTDM Strength
461. wavelet-LH first-order 10Percentile
462. wavelet-LH first-order 90Percentile
463. wavelet-LH first-order Energy
464. wavelet-LH first-order Entropy
465. wavelet-LH first-order Interquartile Range
466. wavelet-LH first-order Kurtosis
467. wavelet-LH first-order Maximum
468. wavelet-LH first-order Mean Absolute Deviation
469. wavelet-LH first-order Mean
470. wavelet-LH first-order Median
471. wavelet-LH first-order Minimum
472. wavelet-LH first-order Range
473. wavelet-LH first-order Robust Mean Absolute Deviation
474. wavelet-LH first-order Root Mean Squared
475. wavelet-LH first-order Skewness
476. wavelet-LH first-order Total Energy
477. wavelet-LH first-order Uniformity
478. wavelet-LH first-order Variance
479. wavelet-LH GLCM Autocorrelation
480. wavelet-LH GLCM Cluster Prominence
481. wavelet-LH GLCM Cluster Shade
482. wavelet-LH GLCM Cluster Tendency
483. wavelet-LH GLCM Contrast
484. wavelet-LH GLCM Correlation
485. wavelet-LH GLCM Difference Average
486. wavelet-LH GLCM Difference Entropy
487. wavelet-LH GLCM Difference Variance
488. wavelet-LH GLCM Inverse Difference
489. wavelet-LH GLCM Inverse Difference Moment
490. wavelet-LH GLCM Inverse Difference Moment Normalized
491. wavelet-LH GLCM Inverse Difference Normalized
492. wavelet-LH GLCM Informational Measure of Correlation 1
493. wavelet-LH GLCM Informational Measure of Correlation 2
494. wavelet-LH GLCM Inverse Variance
495. wavelet-LH GLCM Joint Average
496. wavelet-LH GLCM Joint Energy
497. wavelet-LH GLCM Joint Entropy
498. wavelet-LH GLCM MCC
499. wavelet-LH GLCM Maximum Probability
500. wavelet-LH GLCM Sum Entropy
501. wavelet-LH GLCM Sum Squares
502. wavelet-LH GLRLM Gray Level Non-Uniformity
503. wavelet-LH GLRLM Gray Level Non-Uniformity Normalized
504. wavelet-LH GLRLM Gray Level Variance
505. wavelet-LH GLRLM High Gray Level Run Emphasis
506. wavelet-LH GLRLM Long Run Emphasis
507. wavelet-LH GLRLM Long Run High Gray Level Emphasis
508. wavelet-LH GLRLM Long Run Low Gray Level Emphasis
509. wavelet-LH GLRLM Low Gray Level Run Emphasis
510. wavelet-LH GLRLM Run Entropy
511. wavelet-LH GLRLM Run Length Non-Uniformity
512. wavelet-LH GLRLM Run Length Non-Uniformity Normalized
513. wavelet-LH GLRLM Run Percentage
514. wavelet-LH GLRLM Run Variance
515. wavelet-LH GLRLM Short Run Emphasis
516. wavelet-LH GLRLM Short Run High Gray Level Emphasis
517. wavelet-LH GLRLM Short Run Low Gray Level Emphasis
518. wavelet-LH GLSZM Gray Level Non-Uniformity
519. wavelet-LH GLSZM Gray Level Non-Uniformity Normalized
520. wavelet-LH GLSZM Gray Level Variance
521. wavelet-LH GLSZM High Gray Level Zone Emphasis
522. wavelet-LH GLSZM Large Area Emphasis
523. wavelet-LH GLSZM Large Area High Gray Level Emphasis
524. wavelet-LH GLSZM Large Area Low Gray Level Emphasis
525. wavelet-LH GLSZM Low Gray Level Zone Emphasis
526. wavelet-LH GLSZM Size Zone Non-Uniformity
527. wavelet-LH GLSZM Size Zone Non-Uniformity Normalized
528. wavelet-LH GLSZM Small Area Emphasis
529. wavelet-LH GLSZM Small Area High Gray Level Emphasis
530. wavelet-LH GLSZM Small Area Low Gray Level Emphasis
531. wavelet-LH GLSZM Zone Entropy
532. wavelet-LH GLSZM Zone Percentage
533. wavelet-LH GLSZM Zone Variance
534. wavelet-LH GLDM Dependence Entropy
535. wavelet-LH GLDM Dependence Non-Uniformity
536. wavelet-LH GLDM Dependence Non-Uniformity Normalized
537. wavelet-LH GLDM Dependence Variance
538. wavelet-LH GLDM Gray Level Non-Uniformity
539. wavelet-LH GLDM Gray Level Variance
540. wavelet-LH GLDM High Gray Level Emphasis
541. wavelet-LH GLDM Large Dependence Emphasis
542. wavelet-LH GLDM Large Dependence High Gray Level Emphasis
543. wavelet-LH GLDM Large Dependence Low Gray Level Emphasis
544. wavelet-LH GLDM Low Gray Level Emphasis
545. wavelet-LH GLDM Small Dependence Emphasis
546. wavelet-LH GLDM Small Dependence High Gray Level Emphasis
547. wavelet-LH GLDM Small Dependence Low Gray Level Emphasis
548. wavelet-LH NGTDM Busyness
549. wavelet-LH NGTDM Coarseness
550. wavelet-LH NGTDM Complexity
551. wavelet-LH NGTDM Contrast
552. wavelet-LH NGTDM Strength
553. wavelet-HL first-order 10Percentile
554. wavelet-HL first-order 90Percentile
555. wavelet-HL first-order Energy
556. wavelet-HL first-order Entropy
557. wavelet-HL first-order Interquartile Range
558. wavelet-HL first-order Kurtosis
559. wavelet-HL first-order Maximum
560. wavelet-HL first-order Mean Absolute Deviation
561. wavelet-HL first-order Mean
562. wavelet-HL first-order Median
563. wavelet-HL first-order Minimum
564. wavelet-HL first-order Range
565. wavelet-HL first-order Robust Mean Absolute Deviation
566. wavelet-HL first-order Root Mean Squared
567. wavelet-HL first-order Skewness
568. wavelet-HL first-order Total Energy
569. wavelet-HL first-order Uniformity
570. wavelet-HL first-order Variance
571. wavelet-HL GLCM Autocorrelation
572. wavelet-HL GLCM Cluster Prominence
573. wavelet-HL GLCM Cluster Shade
574. wavelet-HL GLCM Cluster Tendency
575. wavelet-HL GLCM Contrast
576. wavelet-HL GLCM Correlation
577. wavelet-HL GLCM Difference Average
578. wavelet-HL GLCM Difference Entropy
579. wavelet-HL GLCM Difference Variance
580. wavelet-HL GLCM Inverse Difference
581. wavelet-HL GLCM Inverse Difference Moment
582. wavelet-HL GLCM Inverse Difference Moment Normalized
583. wavelet-HL GLCM Inverse Difference Normalized
584. wavelet-HL GLCM Informational Measure of Correlation 1
585. wavelet-HL GLCM Informational Measure of Correlation 2
586. wavelet-HL GLCM Inverse Variance
587. wavelet-HL GLCM Joint Average
588. wavelet-HL GLCM Joint Energy
589. wavelet-HL GLCM Joint Entropy
590. wavelet-HL GLCM MCC
591. wavelet-HL GLCM Maximum Probability
592. wavelet-HL GLCM Sum Entropy
593. wavelet-HL GLCM Sum Squares
594. wavelet-HL GLRLM Gray Level Non-Uniformity
595. wavelet-HL GLRLM Gray Level Non-Uniformity Normalized
596. wavelet-HL GLRLM Gray Level Variance
597. wavelet-HL GLRLM High Gray Level Run Emphasis
598. wavelet-HL GLRLM Long Run Emphasis
599. wavelet-HL GLRLM Long Run High Gray Level Emphasis
600. wavelet-HL GLRLM Long Run Low Gray Level Emphasis
601. wavelet-HL GLRLM Low Gray Level Run Emphasis
602. wavelet-HL GLRLM Run Entropy
603. wavelet-HL GLRLM Run Length Non-Uniformity
604. wavelet-HL GLRLM Run Length Non-Uniformity Normalized
605. wavelet-HL GLRLM Run Percentage
606. wavelet-HL GLRLM Run Variance
607. wavelet-HL GLRLM Short Run Emphasis
608. wavelet-HL GLRLM Short Run High Gray Level Emphasis
609. wavelet-HL GLRLM Short Run Low Gray Level Emphasis
610. wavelet-HL GLSZM Gray Level Non-Uniformity
611. wavelet-HL GLSZM Gray Level Non-Uniformity Normalized
612. wavelet-HL GLSZM Gray Level Variance
613. wavelet-HL GLSZM High Gray Level Zone Emphasis
614. wavelet-HL GLSZM Large Area Emphasis
615. wavelet-HL GLSZM Large Area High Gray Level Emphasis
616. wavelet-HL GLSZM Large Area Low Gray Level Emphasis
617. wavelet-HL GLSZM Low Gray Level Zone Emphasis
618. wavelet-HL GLSZM Size Zone Non-Uniformity
619. wavelet-HL GLSZM Size Zone Non-Uniformity Normalized
620. wavelet-HL GLSZM Small Area Emphasis
621. wavelet-HL GLSZM Small Area High Gray Level Emphasis
622. wavelet-HL GLSZM Small Area Low Gray Level Emphasis
623. wavelet-HL GLSZM Zone Entropy
624. wavelet-HL GLSZM Zone Percentage
625. wavelet-HL GLSZM Zone Variance
626. wavelet-HL GLDM Dependence Entropy
627. wavelet-HL GLDM Dependence Non-Uniformity
628. wavelet-HL GLDM Dependence Non-Uniformity Normalized
629. wavelet-HL GLDM Dependence Variance
630. wavelet-HL GLDM Gray Level Non-Uniformity
631. wavelet-HL GLDM Gray Level Variance
632. wavelet-HL GLDM High Gray Level Emphasis
633. wavelet-HL GLDM Large Dependence Emphasis
634. wavelet-HL GLDM Large Dependence High Gray Level Emphasis
635. wavelet-HL GLDM Large Dependence Low Gray Level Emphasis
636. wavelet-HL GLDM Low Gray Level Emphasis
637. wavelet-HL GLDM Small Dependence Emphasis
638. wavelet-HL GLDM Small Dependence High Gray Level Emphasis
639. wavelet-HL GLDM Small Dependence Low Gray Level Emphasis
640. wavelet-HL NGTDM Busyness
641. wavelet-HL NGTDM Coarseness
642. wavelet-HL NGTDM Complexity
643. wavelet-HL NGTDM Contrast
644. wavelet-HL NGTDM Strength
645. wavelet-HH first-order 10Percentile
646. wavelet-HH first-order 90Percentile
647. wavelet-HH first-order Energy
648. wavelet-HH first-order Entropy
649. wavelet-HH first-order Interquartile Range
650. wavelet-HH first-order Kurtosis
651. wavelet-HH first-order Maximum
652. wavelet-HH first-order Mean Absolute Deviation
653. wavelet-HH first-order Mean
654. wavelet-HH first-order Median
655. wavelet-HH first-order Minimum
656. wavelet-HH first-order Range
657. wavelet-HH first-order Robust Mean Absolute Deviation
658. wavelet-HH first-order Root Mean Squared
659. wavelet-HH first-order Skewness
660. wavelet-HH first-order Total Energy
661. wavelet-HH first-order Uniformity
662. wavelet-HH first-order Variance
663. wavelet-HH GLCM Autocorrelation
664. wavelet-HH GLCM Cluster Prominence
665. wavelet-HH GLCM Cluster Shade
666. wavelet-HH GLCM Cluster Tendency
667. wavelet-HH GLCM Contrast
668. wavelet-HH GLCM Correlation
669. wavelet-HH GLCM Difference Average
670. wavelet-HH GLCM Difference Entropy
671. wavelet-HH GLCM Difference Variance
672. wavelet-HH GLCM Inverse Difference
673. wavelet-HH GLCM Inverse Difference Moment
674. wavelet-HH GLCM Inverse Difference Moment Normalized
675. wavelet-HH GLCM Inverse Difference Normalized
676. wavelet-HH GLCM Informational Measure of Correlation 1
677. wavelet-HH GLCM Informational Measure of Correlation 2
678. wavelet-HH GLCM Inverse Variance
679. wavelet-HH GLCM Joint Average
680. wavelet-HH GLCM Joint Energy
681. wavelet-HH GLCM Joint Entropy
682. wavelet-HH GLCM MCC
683. wavelet-HH GLCM Maximum Probability
684. wavelet-HH GLCM Sum Entropy
685. wavelet-HH GLCM Sum Squares
686. wavelet-HH GLRLM Gray Level Non-Uniformity
687. wavelet-HH GLRLM Gray Level Non-Uniformity Normalized
688. wavelet-HH GLRLM Gray Level Variance
689. wavelet-HH GLRLM High Gray Level Run Emphasis
690. wavelet-HH GLRLM Long Run Emphasis
691. wavelet-HH GLRLM Long Run High Gray Level Emphasis
692. wavelet-HH GLRLM Long Run Low Gray Level Emphasis
693. wavelet-HH GLRLM Low Gray Level Run Emphasis
694. wavelet-HH GLRLM Run Entropy
695. wavelet-HH GLRLM Run Length Non-Uniformity
696. wavelet-HH GLRLM Run Length Non-Uniformity Normalized
697. wavelet-HH GLRLM Run Percentage
698. wavelet-HH GLRLM Run Variance
699. wavelet-HH GLRLM Short Run Emphasis
700. wavelet-HH GLRLM Short Run High Gray Level Emphasis
701. wavelet-HH GLRLM Short Run Low Gray Level Emphasis
702. wavelet-HH GLSZM Gray Level Non-Uniformity
703. wavelet-HH GLSZM Gray Level Non-Uniformity Normalized
704. wavelet-HH GLSZM Gray Level Variance
705. wavelet-HH GLSZM High Gray Level Zone Emphasis
706. wavelet-HH GLSZM Large Area Emphasis
707. wavelet-HH GLSZM Large Area High Gray Level Emphasis
708. wavelet-HH GLSZM Large Area Low Gray Level Emphasis
709. wavelet-HH GLSZM Low Gray Level Zone Emphasis
710. wavelet-HH GLSZM Size Zone Non-Uniformity
711. wavelet-HH GLSZM Size Zone Non-Uniformity Normalized
712. wavelet-HH GLSZM Small Area Emphasis
713. wavelet-HH GLSZM Small Area High Gray Level Emphasis
714. wavelet-HH GLSZM Small Area Low Gray Level Emphasis
715. wavelet-HH GLSZM Zone Entropy
716. wavelet-HH GLSZM Zone Percentage
717. wavelet-HH GLSZM Zone Variance
718. wavelet-HH GLDM Dependence Entropy
719. wavelet-HH GLDM Dependence Non-Uniformity
720. wavelet-HH GLDM Dependence Non-Uniformity Normalized
721. wavelet-HH GLDM Dependence Variance
722. wavelet-HH GLDM Gray Level Non-Uniformity
723. wavelet-HH GLDM Gray Level Variance
724. wavelet-HH GLDM High Gray Level Emphasis
725. wavelet-HH GLDM Large Dependence Emphasis
726. wavelet-HH GLDM Large Dependence High Gray Level Emphasis
727. wavelet-HH GLDM Large Dependence Low Gray Level Emphasis
728. wavelet-HH GLDM Low Gray Level Emphasis
729. wavelet-HH GLDM Small Dependence Emphasis
730. wavelet-HH GLDM Small Dependence High Gray Level Emphasis
731. wavelet-HH GLDM Small Dependence Low Gray Level Emphasis
732. wavelet-HH NGTDM Busyness
733. wavelet-HH NGTDM Coarseness
734. wavelet-HH NGTDM Complexity
735. wavelet-HH NGTDM Contrast
736. wavelet-HH NGTDM Strength
737. wavelet-LL first-order 10Percentile
738. wavelet-LL first-order 90Percentile
739. wavelet-LL first-order Energy
740. wavelet-LL first-order Entropy
741. wavelet-LL first-order Interquartile Range
742. wavelet-LL first-order Kurtosis
743. wavelet-LL first-order Maximum
744. wavelet-LL first-order Mean Absolute Deviation
745. wavelet-LL first-order Mean
746. wavelet-LL first-order Median
747. wavelet-LL first-order Minimum
748. wavelet-LL first-order Range
749. wavelet-LL first-order Robust Mean Absolute Deviation
750. wavelet-LL first-order Root Mean Squared
751. wavelet-LL first-order Skewness
752. wavelet-LL first-order Total Energy
753. wavelet-LL first-order Uniformity
754. wavelet-LL first-order Variance
755. wavelet-LL GLCM Autocorrelation
756. wavelet-LL GLCM Cluster Prominence
757. wavelet-LL GLCM Cluster Shade
758. wavelet-LL GLCM Cluster Tendency
759. wavelet-LL GLCM Contrast
760. wavelet-LL GLCM Correlation
761. wavelet-LL GLCM Difference Average
762. wavelet-LL GLCM Difference Entropy
763. wavelet-LL GLCM Difference Variance
764. wavelet-LL GLCM Inverse Difference
765. wavelet-LL GLCM Inverse Difference Moment
766. wavelet-LL GLCM Inverse Difference Moment Normalized
767. wavelet-LL GLCM Inverse Difference Normalized
768. wavelet-LL GLCM Informational Measure of Correlation 1
769. wavelet-LL GLCM Informational Measure of Correlation 2
770. wavelet-LL GLCM Inverse Variance
771. wavelet-LL GLCM Joint Average
772. wavelet-LL GLCM Joint Energy
773. wavelet-LL GLCM Joint Entropy
774. wavelet-LL GLCM MCC
775. wavelet-LL GLCM Maximum Probability
776. wavelet-LL GLCM Sum Entropy
777. wavelet-LL GLCM Sum Squares
778. wavelet-LL GLRLM Gray Level Non-Uniformity
779. wavelet-LL GLRLM Gray Level Non-Uniformity Normalized
780. wavelet-LL GLRLM Gray Level Variance
781. wavelet-LL GLRLM High Gray Level Run Emphasis
782. wavelet-LL GLRLM Long Run Emphasis
783. wavelet-LL GLRLM Long Run High Gray Level Emphasis
784. wavelet-LL GLRLM Long Run Low Gray Level Emphasis
785. wavelet-LL GLRLM Low Gray Level Run Emphasis
786. wavelet-LL GLRLM Run Entropy
787. wavelet-LL GLRLM Run Length Non-Uniformity
788. wavelet-LL GLRLM Run Length Non-Uniformity Normalized
789. wavelet-LL GLRLM Run Percentage
790. wavelet-LL GLRLM Run Variance
791. wavelet-LL GLRLM Short Run Emphasis
792. wavelet-LL GLRLM Short Run High Gray Level Emphasis
793. wavelet-LL GLRLM Short Run Low Gray Level Emphasis
794. wavelet-LL GLSZM Gray Level Non-Uniformity
795. wavelet-LL GLSZM Gray Level Non-Uniformity Normalized
796. wavelet-LL GLSZM Gray Level Variance
797. wavelet-LL GLSZM High Gray Level Zone Emphasis
798. wavelet-LL GLSZM Large Area Emphasis
799. wavelet-LL GLSZM Large Area High Gray Level Emphasis
800. wavelet-LL GLSZM Large Area Low Gray Level Emphasis
801. wavelet-LL GLSZM Low Gray Level Zone Emphasis
802. wavelet-LL GLSZM Size Zone Non-Uniformity
803. wavelet-LL GLSZM Size Zone Non-Uniformity Normalized
804. wavelet-LL GLSZM Small Area Emphasis
805. wavelet-LL GLSZM Small Area High Gray Level Emphasis
806. wavelet-LL GLSZM Small Area Low Gray Level Emphasis
807. wavelet-LL GLSZM Zone Entropy
808. wavelet-LL GLSZM Zone Percentage
809. wavelet-LL GLSZM Zone Variance
810. wavelet-LL GLDM Dependence Entropy
811. wavelet-LL GLDM Dependence Non-Uniformity
812. wavelet-LL GLDM Dependence Non-Uniformity Normalized
813. wavelet-LL GLDM Dependence Variance
814. wavelet-LL GLDM Gray Level Non-Uniformity
815. wavelet-LL GLDM Gray Level Variance
816. wavelet-LL GLDM High Gray Level Emphasis
817. wavelet-LL GLDM Large Dependence Emphasis
818. wavelet-LL GLDM Large Dependence High Gray Level Emphasis
819. wavelet-LL GLDM Large Dependence Low Gray Level Emphasis
820. wavelet-LL GLDM Low Gray Level Emphasis
821. wavelet-LL GLDM Small Dependence Emphasis
822. wavelet-LL GLDM Small Dependence High Gray Level Emphasis
823. wavelet-LL GLDM Small Dependence Low Gray Level Emphasis
824. wavelet-LL NGTDM Busyness
825. wavelet-LL NGTDM Coarseness
826. wavelet-LL NGTDM Complexity
827. wavelet-LL NGTDM Contrast
828. wavelet-LL NGTDM Strength
829. square first-order 10Percentile
830. square first-order 90Percentile
831. square first-order Energy
832. square first-order Entropy
833. square first-order Interquartile Range
834. square first-order Kurtosis
835. square first-order Maximum
836. square first-order Mean Absolute Deviation
837. square first-order Mean
838. square first-order Median
839. square first-order Minimum
840. square first-order Range
841. square first-order Robust Mean Absolute Deviation
842. square first-order Root Mean Squared
843. square first-order Skewness
844. square first-order Total Energy
845. square first-order Uniformity
846. square first-order Variance
847. square GLCM Autocorrelation
848. square GLCM Cluster Prominence
849. square GLCM Cluster Shade
850. square GLCM Cluster Tendency
851. square GLCM Contrast
852. square GLCM Correlation
853. square GLCM Difference Average
854. square GLCM Difference Entropy
855. square GLCM Difference Variance
856. square GLCM Inverse Difference
857. square GLCM Inverse Difference Moment
858. square GLCM Inverse Difference Moment Normalized
859. square GLCM Inverse Difference Normalized
860. square GLCM Informational Measure of Correlation 1
861. square GLCM Informational Measure of Correlation 2
862. square GLCM Inverse Variance
863. square GLCM Joint Average
864. square GLCM Joint Energy
865. square GLCM Joint Entropy
866. square GLCM MCC
867. square GLCM Maximum Probability
868. square GLCM Sum Entropy
869. square GLCM Sum Squares
870. square GLRLM Gray Level Non-Uniformity
871. square GLRLM Gray Level Non-Uniformity Normalized
872. square GLRLM Gray Level Variance
873. square GLRLM High Gray Level Run Emphasis
874. square GLRLM Long Run Emphasis
875. square GLRLM Long Run High Gray Level Emphasis
876. square GLRLM Long Run Low Gray Level Emphasis
877. square GLRLM Low Gray Level Run Emphasis
878. square GLRLM Run Entropy
879. square GLRLM Run Length Non-Uniformity
880. square GLRLM Run Length Non-Uniformity Normalized
881. square GLRLM Run Percentage
882. square GLRLM Run Variance
883. square GLRLM Short Run Emphasis
884. square GLRLM Short Run High Gray Level Emphasis
885. square GLRLM Short Run Low Gray Level Emphasis
886. square GLSZM Gray Level Non-Uniformity
887. square GLSZM Gray Level Non-Uniformity Normalized
888. square GLSZM Gray Level Variance
889. square GLSZM High Gray Level Zone Emphasis
890. square GLSZM Large Area Emphasis
891. square GLSZM Large Area High Gray Level Emphasis
892. square GLSZM Large Area Low Gray Level Emphasis
893. square GLSZM Low Gray Level Zone Emphasis
894. square GLSZM Size Zone Non-Uniformity
895. square GLSZM Size Zone Non-Uniformity Normalized
896. square GLSZM Small Area Emphasis
897. square GLSZM Small Area High Gray Level Emphasis
898. square GLSZM Small Area Low Gray Level Emphasis
899. square GLSZM Zone Entropy
900. square GLSZM Zone Percentage
901. square GLSZM Zone Variance
902. square GLDM Dependence Entropy
903. square GLDM Dependence Non-Uniformity
904. square GLDM Dependence Non-Uniformity Normalized
905. square GLDM Dependence Variance
906. square GLDM Gray Level Non-Uniformity
907. square GLDM Gray Level Variance
908. square GLDM High Gray Level Emphasis
909. square GLDM Large Dependence Emphasis
910. square GLDM Large Dependence High Gray Level Emphasis
911. square GLDM Large Dependence Low Gray Level Emphasis
912. square GLDM Low Gray Level Emphasis
913. square GLDM Small Dependence Emphasis
914. square GLDM Small Dependence High Gray Level Emphasis
915. square GLDM Small Dependence Low Gray Level Emphasis
916. square NGTDM Busyness
917. square NGTDM Coarseness
918. square NGTDM Complexity
919. square NGTDM Contrast
920. square NGTDM Strength
921. squareroot first-order 10Percentile
922. squareroot first-order 90Percentile
923. squareroot first-order Energy
924. squareroot first-order Entropy
925. squareroot first-order Interquartile Range
926. squareroot first-order Kurtosis
927. squareroot first-order Maximum
928. squareroot first-order Mean Absolute Deviation
929. squareroot first-order Mean
930. squareroot first-order Median
931. squareroot first-order Minimum
932. squareroot first-order Range
933. squareroot first-order Robust Mean Absolute Deviation
934. squareroot first-order Root Mean Squared
935. squareroot first-order Skewness
936. squareroot first-order Total Energy
937. squareroot first-order Uniformity
938. squareroot first-order Variance
939. squareroot GLCM Autocorrelation
940. squareroot GLCM Cluster Prominence
941. squareroot GLCM Cluster Shade
942. squareroot GLCM Cluster Tendency
943. squareroot GLCM Contrast
944. squareroot GLCM Correlation
945. squareroot GLCM Difference Average
946. squareroot GLCM Difference Entropy
947. squareroot GLCM Difference Variance
948. squareroot GLCM Inverse Difference
949. squareroot GLCM Inverse Difference Moment
950. squareroot GLCM Inverse Difference Moment Normalized
951. squareroot GLCM Inverse Difference Normalized
952. squareroot GLCM Informational Measure of Correlation 1
953. squareroot GLCM Informational Measure of Correlation 2
954. squareroot GLCM Inverse Variance
955. squareroot GLCM Joint Average
956. squareroot GLCM Joint Energy
957. squareroot GLCM Joint Entropy
958. squareroot GLCM MCC
959. squareroot GLCM Maximum Probability
960. squareroot GLCM Sum Entropy
961. squareroot GLCM Sum Squares
962. squareroot GLRLM Gray Level Non-Uniformity
963. squareroot GLRLM Gray Level Non-Uniformity Normalized
964. squareroot GLRLM Gray Level Variance
965. squareroot GLRLM High Gray Level Run Emphasis
966. squareroot GLRLM Long Run Emphasis
967. squareroot GLRLM Long Run High Gray Level Emphasis
968. squareroot GLRLM Long Run Low Gray Level Emphasis
969. squareroot GLRLM Low Gray Level Run Emphasis
970. squareroot GLRLM Run Entropy
971. squareroot GLRLM Run Length Non-Uniformity
972. squareroot GLRLM Run Length Non-Uniformity Normalized
973. squareroot GLRLM Run Percentage
974. squareroot GLRLM Run Variance
975. squareroot GLRLM Short Run Emphasis
976. squareroot GLRLM Short Run High Gray Level Emphasis
977. squareroot GLRLM Short Run Low Gray Level Emphasis
978. squareroot GLSZM Gray Level Non-Uniformity
979. squareroot GLSZM Gray Level Non-Uniformity Normalized
980. squareroot GLSZM Gray Level Variance
981. squareroot GLSZM High Gray Level Zone Emphasis
982. squareroot GLSZM Large Area Emphasis
983. squareroot GLSZM Large Area High Gray Level Emphasis
984. squareroot GLSZM Large Area Low Gray Level Emphasis
985. squareroot GLSZM Low Gray Level Zone Emphasis
986. squareroot GLSZM Size Zone Non-Uniformity
987. squareroot GLSZM Size Zone Non-Uniformity Normalized
988. squareroot GLSZM Small Area Emphasis
989. squareroot GLSZM Small Area High Gray Level Emphasis
990. squareroot GLSZM Small Area Low Gray Level Emphasis
991. squareroot GLSZM Zone Entropy
992. squareroot GLSZM Zone Percentage
993. squareroot GLSZM Zone Variance
994. squareroot GLDM Dependence Entropy
995. squareroot GLDM Dependence Non-Uniformity
996. squareroot GLDM Dependence Non-Uniformity Normalized
997. squareroot GLDM Dependence Variance
998. squareroot GLDM Gray Level Non-Uniformity
999. squareroot GLDM Gray Level Variance
1000. squareroot GLDM High Gray Level Emphasis
1001. squareroot GLDM Large Dependence Emphasis
1002. squareroot GLDM Large Dependence High Gray Level Emphasis
1003. squareroot GLDM Large Dependence Low Gray Level Emphasis
1004. squareroot GLDM Low Gray Level Emphasis
1005. squareroot GLDM Small Dependence Emphasis
1006. squareroot GLDM Small Dependence High Gray Level Emphasis
1007. squareroot GLDM Small Dependence Low Gray Level Emphasis
1008. squareroot NGTDM Busyness
1009. squareroot NGTDM Coarseness
1010. squareroot NGTDM Complexity
1011. squareroot NGTDM Contrast
1012. squareroot NGTDM Strength
1013. logarithm first-order 10Percentile
1014. logarithm first-order 90Percentile
1015. logarithm first-order Energy
1016. logarithm first-order Entropy
1017. logarithm first-order Interquartile Range
1018. logarithm first-order Kurtosis
1019. logarithm first-order Maximum
1020. logarithm first-order Mean Absolute Deviation
1021. logarithm first-order Mean
1022. logarithm first-order Median
1023. logarithm first-order Minimum
1024. logarithm first-order Range
1025. logarithm first-order Robust Mean Absolute Deviation
1026. logarithm first-order Root Mean Squared
1027. logarithm first-order Skewness
1028. logarithm first-order Total Energy
1029. logarithm first-order Uniformity
1030. logarithm first-order Variance
1031. logarithm GLCM Autocorrelation
1032. logarithm GLCM Cluster Prominence
1033. logarithm GLCM Cluster Shade
1034. logarithm GLCM Cluster Tendency
1035. logarithm GLCM Contrast
1036. logarithm GLCM Correlation
1037. logarithm GLCM Difference Average
1038. logarithm GLCM Difference Entropy
1039. logarithm GLCM Difference Variance
1040. logarithm GLCM Inverse Difference
1041. logarithm GLCM Inverse Difference Moment
1042. logarithm GLCM Inverse Difference Moment Normalized
1043. logarithm GLCM Inverse Difference Normalized
1044. logarithm GLCM Informational Measure of Correlation 1
1045. logarithm GLCM Informational Measure of Correlation 2
1046. logarithm GLCM Inverse Variance
1047. logarithm GLCM Joint Average
1048. logarithm GLCM Joint Energy
1049. logarithm GLCM Joint Entropy
1050. logarithm GLCM MCC
1051. logarithm GLCM Maximum Probability
1052. logarithm GLCM Sum Entropy
1053. logarithm GLCM Sum Squares
1054. logarithm GLRLM Gray Level Non-Uniformity
1055. logarithm GLRLM Gray Level Non-Uniformity Normalized
1056. logarithm GLRLM Gray Level Variance
1057. logarithm GLRLM High Gray Level Run Emphasis
1058. logarithm GLRLM Long Run Emphasis
1059. logarithm GLRLM Long Run High Gray Level Emphasis
1060. logarithm GLRLM Long Run Low Gray Level Emphasis
1061. logarithm GLRLM Low Gray Level Run Emphasis
1062. logarithm GLRLM Run Entropy
1063. logarithm GLRLM Run Length Non-Uniformity
1064. logarithm GLRLM Run Length Non-Uniformity Normalized
1065. logarithm GLRLM Run Percentage
1066. logarithm GLRLM Run Variance
1067. logarithm GLRLM Short Run Emphasis
1068. logarithm GLRLM Short Run High Gray Level Emphasis
1069. logarithm GLRLM Short Run Low Gray Level Emphasis
1070. logarithm GLSZM Gray Level Non-Uniformity
1071. logarithm GLSZM Gray Level Non-Uniformity Normalized
1072. logarithm GLSZM Gray Level Variance
1073. logarithm GLSZM High Gray Level Zone Emphasis
1074. logarithm GLSZM Large Area Emphasis
1075. logarithm GLSZM Large Area High Gray Level Emphasis
1076. logarithm GLSZM Large Area Low Gray Level Emphasis
1077. logarithm GLSZM Low Gray Level Zone Emphasis
1078. logarithm GLSZM Size Zone Non-Uniformity
1079. logarithm GLSZM Size Zone Non-Uniformity Normalized
1080. logarithm GLSZM Small Area Emphasis
1081. logarithm GLSZM Small Area High Gray Level Emphasis
1082. logarithm GLSZM Small Area Low Gray Level Emphasis
1083. logarithm GLSZM Zone Entropy
1084. logarithm GLSZM Zone Percentage
1085. logarithm GLSZM Zone Variance
1086. logarithm GLDM Dependence Entropy
1087. logarithm GLDM Dependence Non-Uniformity
1088. logarithm GLDM Dependence Non-Uniformity Normalized
1089. logarithm GLDM Dependence Variance
1090. logarithm GLDM Gray Level Non-Uniformity
1091. logarithm GLDM Gray Level Variance
1092. logarithm GLDM High Gray Level Emphasis
1093. logarithm GLDM Large Dependence Emphasis
1094. logarithm GLDM Large Dependence High Gray Level Emphasis
1095. logarithm GLDM Large Dependence Low Gray Level Emphasis
1096. logarithm GLDM Low Gray Level Emphasis
1097. logarithm GLDM Small Dependence Emphasis
1098. logarithm GLDM Small Dependence High Gray Level Emphasis
1099. logarithm GLDM Small Dependence Low Gray Level Emphasis
1100. logarithm NGTDM Busyness
1101. logarithm NGTDM Coarseness
1102. logarithm NGTDM Complexity
1103. logarithm NGTDM Contrast
1104. logarithm NGTDM Strength
1105. exponential first-order 10Percentile
1106. exponential first-order 90Percentile
1107. exponential first-order Energy
1108. exponential first-order Entropy
1109. exponential first-order Interquartile Range
1110. exponential first-order Kurtosis
1111. exponential first-order Maximum
1112. exponential first-order Mean Absolute Deviation
1113. exponential first-order Mean
1114. exponential first-order Median
1115. exponential first-order Minimum
1116. exponential first-order Range
1117. exponential first-order Robust Mean Absolute Deviation
1118. exponential first-order Root Mean Squared
1119. exponential first-order Skewness
1120. exponential first-order Total Energy
1121. exponential first-order Uniformity
1122. exponential first-order Variance
1123. exponential GLCM Autocorrelation
1124. exponential GLCM Cluster Prominence
1125. exponential GLCM Cluster Shade
1126. exponential GLCM Cluster Tendency
1127. exponential GLCM Contrast
1128. exponential GLCM Correlation
1129. exponential GLCM Difference Average
1130. exponential GLCM Difference Entropy
1131. exponential GLCM Difference Variance
1132. exponential GLCM Inverse Difference
1133. exponential GLCM Inverse Difference Moment
1134. exponential GLCM Inverse Difference Moment Normalized
1135. exponential GLCM Inverse Difference Normalized
1136. exponential GLCM Informational Measure of Correlation 1
1137. exponential GLCM Informational Measure of Correlation 2
1138. exponential GLCM Inverse Variance
1139. exponential GLCM Joint Average
1140. exponential GLCM Joint Energy
1141. exponential GLCM Joint Entropy
1142. exponential GLCM MCC
1143. exponential GLCM Maximum Probability
1144. exponential GLCM Sum Entropy
1145. exponential GLCM Sum Squares
1146. exponential GLRLM Gray Level Non-Uniformity
1147. exponential GLRLM Gray Level Non-Uniformity Normalized
1148. exponential GLRLM Gray Level Variance
1149. exponential GLRLM High Gray Level Run Emphasis
1150. exponential GLRLM Long Run Emphasis
1151. exponential GLRLM Long Run High Gray Level Emphasis
1152. exponential GLRLM Long Run Low Gray Level Emphasis
1153. exponential GLRLM Low Gray Level Run Emphasis
1154. exponential GLRLM Run Entropy
1155. exponential GLRLM Run Length Non-Uniformity
1156. exponential GLRLM Run Length Non-Uniformity Normalized
1157. exponential GLRLM Run Percentage
1158. exponential GLRLM Run Variance
1159. exponential GLRLM Short Run Emphasis
1160. exponential GLRLM Short Run High Gray Level Emphasis
1161. exponential GLRLM Short Run Low Gray Level Emphasis
1162. exponential GLSZM Gray Level Non-Uniformity
1163. exponential GLSZM Gray Level Non-Uniformity Normalized
1164. exponential GLSZM Gray Level Variance
1165. exponential GLSZM High Gray Level Zone Emphasis
1166. exponential GLSZM Large Area Emphasis
1167. exponential GLSZM Large Area High Gray Level Emphasis
1168. exponential GLSZM Large Area Low Gray Level Emphasis
1169. exponential GLSZM Low Gray Level Zone Emphasis
1170. exponential GLSZM Size Zone Non-Uniformity
1171. exponential GLSZM Size Zone Non-Uniformity Normalized
1172. exponential GLSZM Small Area Emphasis
1173. exponential GLSZM Small Area High Gray Level Emphasis
1174. exponential GLSZM Small Area Low Gray Level Emphasis
1175. exponential GLSZM Zone Entropy
1176. exponential GLSZM Zone Percentage
1177. exponential GLSZM Zone Variance
1178. exponential GLDM Dependence Entropy
1179. exponential GLDM Dependence Non-Uniformity
1180. exponential GLDM Dependence Non-Uniformity Normalized
1181. exponential GLDM Dependence Variance
1182. exponential GLDM Gray Level Non-Uniformity
1183. exponential GLDM Gray Level Variance
1184. exponential GLDM High Gray Level Emphasis
1185. exponential GLDM Large Dependence Emphasis
1186. exponential GLDM Large Dependence High Gray Level Emphasis
1187. exponential GLDM Large Dependence Low Gray Level Emphasis
1188. exponential GLDM Low Gray Level Emphasis
1189. exponential GLDM Small Dependence Emphasis
1190. exponential GLDM Small Dependence High Gray Level Emphasis
1191. exponential GLDM Small Dependence Low Gray Level Emphasis
1192. exponential NGTDM Busyness
1193. exponential NGTDM Coarseness
1194. exponential NGTDM Complexity
1195. exponential NGTDM Contrast
1196. exponential NGTDM Strength
1197. gradient first-order 10Percentile
1198. gradient first-order 90Percentile
1199. gradient first-order Energy
1200. gradient first-order Entropy
1201. gradient first-order Interquartile Range
1202. gradient first-order Kurtosis
1203. gradient first-order Maximum
1204. gradient first-order Mean Absolute Deviation
1205. gradient first-order Mean
1206. gradient first-order Median
1207. gradient first-order Minimum
1208. gradient first-order Range
1209. gradient first-order Robust Mean Absolute Deviation
1210. gradient first-order Root Mean Squared
1211. gradient first-order Skewness
1212. gradient first-order Total Energy
1213. gradient first-order Uniformity
1214. gradient first-order Variance
1215. gradient GLCM Autocorrelation
1216. gradient GLCM Cluster Prominence
1217. gradient GLCM Cluster Shade
1218. gradient GLCM Cluster Tendency
1219. gradient GLCM Contrast
1220. gradient GLCM Correlation
1221. gradient GLCM Difference Average
1222. gradient GLCM Difference Entropy
1223. gradient GLCM Difference Variance
1224. gradient GLCM Inverse Difference
1225. gradient GLCM Inverse Difference Moment
1226. gradient GLCM Inverse Difference Moment Normalized
1227. gradient GLCM Inverse Difference Normalized
1228. gradient GLCM Informational Measure of Correlation 1
1229. gradient GLCM Informational Measure of Correlation 2
1230. gradient GLCM Inverse Variance
1231. gradient GLCM Joint Average
1232. gradient GLCM Joint Energy
1233. gradient GLCM Joint Entropy
1234. gradient GLCM MCC
1235. gradient GLCM Maximum Probability
1236. gradient GLCM Sum Entropy
1237. gradient GLCM Sum Squares
1238. gradient GLRLM Gray Level Non-Uniformity
1239. gradient GLRLM Gray Level Non-Uniformity Normalized
1240. gradient GLRLM Gray Level Variance
1241. gradient GLRLM High Gray Level Run Emphasis
1242. gradient GLRLM Long Run Emphasis
1243. gradient GLRLM Long Run High Gray Level Emphasis
1244. gradient GLRLM Long Run Low Gray Level Emphasis
1245. gradient GLRLM Low Gray Level Run Emphasis
1246. gradient GLRLM Run Entropy
1247. gradient GLRLM Run Length Non-Uniformity
1248. gradient GLRLM Run Length Non-Uniformity Normalized
1249. gradient GLRLM Run Percentage
1250. gradient GLRLM Run Variance
1251. gradient GLRLM Short Run Emphasis
1252. gradient GLRLM Short Run High Gray Level Emphasis
1253. gradient GLRLM Short Run Low Gray Level Emphasis
1254. gradient GLSZM Gray Level Non-Uniformity
1255. gradient GLSZM Gray Level Non-Uniformity Normalized
1256. gradient GLSZM Gray Level Variance
1257. gradient GLSZM High Gray Level Zone Emphasis
1258. gradient GLSZM Large Area Emphasis
1259. gradient GLSZM Large Area High Gray Level Emphasis
1260. gradient GLSZM Large Area Low Gray Level Emphasis
1261. gradient GLSZM Low Gray Level Zone Emphasis
1262. gradient GLSZM Size Zone Non-Uniformity
1263. gradient GLSZM Size Zone Non-Uniformity Normalized
1264. gradient GLSZM Small Area Emphasis
1265. gradient GLSZM Small Area High Gray Level Emphasis
1266. gradient GLSZM Small Area Low Gray Level Emphasis
1267. gradient GLSZM Zone Entropy
1268. gradient GLSZM Zone Percentage
1269. gradient GLSZM Zone Variance
1270. gradient GLDM Dependence Entropy
1271. gradient GLDM Dependence Non-Uniformity
1272. gradient GLDM Dependence Non-Uniformity Normalized
1273. gradient GLDM Dependence Variance
1274. gradient GLDM Gray Level Non-Uniformity
1275. gradient GLDM Gray Level Variance
1276. gradient GLDM High Gray Level Emphasis
1277. gradient GLDM Large Dependence Emphasis
1278. gradient GLDM Large Dependence High Gray Level Emphasis
1279. gradient GLDM Large Dependence Low Gray Level Emphasis
1280. gradient GLDM Low Gray Level Emphasis
1281. gradient GLDM Small Dependence Emphasis
1282. gradient GLDM Small Dependence High Gray Level Emphasis
1283. gradient GLDM Small Dependence Low Gray Level Emphasis
1284. gradient NGTDM Busyness
1285. gradient NGTDM Coarseness
1286. gradient NGTDM Complexity
1287. gradient NGTDM Contrast
1288. gradient NGTDM Strength
1289. lbp-2D first-order 10Percentile
1290. lbp-2D first-order 90Percentile
1291. lbp-2D first-order Energy
1292. lbp-2D first-order Entropy
1293. lbp-2D first-order Interquartile Range
1294. lbp-2D first-order Kurtosis
1295. lbp-2D first-order Maximum
1296. lbp-2D first-order Mean Absolute Deviation
1297. lbp-2D first-order Mean
1298. lbp-2D first-order Median
1299. lbp-2D first-order Minimum
1300. lbp-2D first-order Range
1301. lbp-2D first-order Robust Mean Absolute Deviation
1302. lbp-2D first-order Root Mean Squared
1303. lbp-2D first-order Skewness
1304. lbp-2D first-order Total Energy
1305. lbp-2D first-order Uniformity
1306. lbp-2D first-order Variance
1307. lbp-2D GLCM Autocorrelation
1308. lbp-2D GLCM Cluster Prominence
1309. lbp-2D GLCM Cluster Shade
1310. lbp-2D GLCM Cluster Tendency
1311. lbp-2D GLCM Contrast
1312. lbp-2D GLCM Correlation
1313. lbp-2D GLCM Difference Average
1314. lbp-2D GLCM Difference Entropy
1315. lbp-2D GLCM Difference Variance
1316. lbp-2D GLCM Inverse Difference
1317. lbp-2D GLCM Inverse Difference Moment
1318. lbp-2D GLCM Inverse Difference Moment Normalized
1319. lbp-2D GLCM Inverse Difference Normalized
1320. lbp-2D GLCM Informational Measure of Correlation 1
1321. lbp-2D GLCM Informational Measure of Correlation 2
1322. lbp-2D GLCM Inverse Variance
1323. lbp-2D GLCM Joint Average
1324. lbp-2D GLCM Joint Energy
1325. lbp-2D GLCM Joint Entropy
1326. lbp-2D GLCM MCC
1327. lbp-2D GLCM Maximum Probability
1328. lbp-2D GLCM Sum Entropy
1329. lbp-2D GLCM Sum Squares
1330. lbp-2D GLRLM Gray Level Non-Uniformity
1331. lbp-2D GLRLM Gray Level Non-Uniformity Normalized
1332. lbp-2D GLRLM Gray Level Variance
1333. lbp-2D GLRLM High Gray Level Run Emphasis
1334. lbp-2D GLRLM Long Run Emphasis
1335. lbp-2D GLRLM Long Run High Gray Level Emphasis
1336. lbp-2D GLRLM Long Run Low Gray Level Emphasis
1337. lbp-2D GLRLM Low Gray Level Run Emphasis
1338. lbp-2D GLRLM Run Entropy
1339. lbp-2D GLRLM Run Length Non-Uniformity
1340. lbp-2D GLRLM Run Length Non-Uniformity Normalized
1341. lbp-2D GLRLM Run Percentage
1342. lbp-2D GLRLM Run Variance
1343. lbp-2D GLRLM Short Run Emphasis
1344. lbp-2D GLRLM Short Run High Gray Level Emphasis
1345. lbp-2D GLRLM Short Run Low Gray Level Emphasis
1346. lbp-2D GLSZM Gray Level Non-Uniformity
1347. lbp-2D GLSZM Gray Level Non-Uniformity Normalized
1348. lbp-2D GLSZM Gray Level Variance
1349. lbp-2D GLSZM High Gray Level Zone Emphasis
1350. lbp-2D GLSZM Large Area Emphasis
1351. lbp-2D GLSZM Large Area High Gray Level Emphasis
1352. lbp-2D GLSZM Large Area Low Gray Level Emphasis
1353. lbp-2D GLSZM Low Gray Level Zone Emphasis
1354. lbp-2D GLSZM Size Zone Non-Uniformity
1355. lbp-2D GLSZM Size Zone Non-Uniformity Normalized
1356. lbp-2D GLSZM Small Area Emphasis
1357. lbp-2D GLSZM Small Area High Gray Level Emphasis
1358. lbp-2D GLSZM Small Area Low Gray Level Emphasis
1359. lbp-2D GLSZM Zone Entropy
1360. lbp-2D GLSZM Zone Percentage
1361. lbp-2D GLSZM Zone Variance
1362. lbp-2D GLDM Dependence Entropy
1363. lbp-2D GLDM Dependence Non-Uniformity
1364. lbp-2D GLDM Dependence Non-Uniformity Normalized
1365. lbp-2D GLDM Dependence Variance
1366. lbp-2D GLDM Gray Level Non-Uniformity
1367. lbp-2D GLDM Gray Level Variance
1368. lbp-2D GLDM High Gray Level Emphasis
1369. lbp-2D GLDM Large Dependence Emphasis
1370. lbp-2D GLDM Large Dependence High Gray Level Emphasis
1371. lbp-2D GLDM Large Dependence Low Gray Level Emphasis
1372. lbp-2D GLDM Low Gray Level Emphasis
1373. lbp-2D GLDM Small Dependence Emphasis
1374. lbp-2D GLDM Small Dependence High Gray Level Emphasis
1375. lbp-2D GLDM Small Dependence Low Gray Level Emphasis
1376. lbp-2D NGTDM Busyness
1377. lbp-2D NGTDM Coarseness
1378. lbp-2D NGTDM Complexity
1379. lbp-2D NGTDM Contrast
1380. lbp-2D NGTDM Strength

## Supplementary Material References:

1. Pedregosa F, Varoquaux G, Gramfort A, Michel V, Thirion B, Grisel O, et al. Scikit-learn: Machine Learning in Python. Journal of Machine Learning Research. 2011;12:2825-30.

2. Lemaitre GaN, Fernando and Aridas, Christos K. Imbalanced-learn: A Python Toolbox to Tackle the Curse of Imbalanced Datasets in Machine Learning. arXiv; 2016.

3. Pilnenskiy NaSI. Feature Selection Algorithms as One of the Python Data Analytical Tools. Future Internet. 2020;12:54.

4. Radovic M, Ghalwash M, Filipovic N, Obradovic Z. Minimum redundancy maximum relevance feature selection approach for temporal gene expression data. BMC Bioinformatics. 2017;18(1):9.

5. De Jay N, Papillon-Cavanagh S, Olsen C, El-Hachem N, Bontempi G, Haibe-Kains B. mRMRe: an R package for parallelized mRMR ensemble feature selection. Bioinformatics. 2013;29(18):2365-8.

6. Parmar C, Grossmann P, Bussink J, Lambin P, Aerts HJWL. Machine Learning methods for Quantitative Radiomic Biomarkers. Sci Rep. 2015;5:13087.

7. Tixier F, Le Rest CC, Hatt M, Albarghach N, Pradier O, Metges JP, et al. Intratumor heterogeneity characterized by textural features on baseline 18F-FDG PET images predicts response to concomitant radiochemotherapy in esophageal cancer. J Nucl Med. 2011;52(3):369-78.
